# Anti-Atherogenic Actions of Pomegranate Polyphenol Punicalagin and Its Metabolites: In Vitro Effects on Vascular Cells and In Vivo Atheroprotection by Urolithin A via Anti-Inflammatory and Plaque-Stabilising Mechanisms

**DOI:** 10.3390/antiox15040507

**Published:** 2026-04-20

**Authors:** Sulaiman Alalawi, Daniah Rifqi, Alaa Alhamadi, Reem Alotibi, Fahad Alradi, Nouf Alshehri, Yee-Hung Chan, Jing Chen, Faizah Albalawi, Sarab Taha, Nabras Al-Mahrami, Irina A. Guschina, Timothy R. Hughes, Dipak P. Ramji

**Affiliations:** 1 Cardiff School of Biosciences, Cardiff University, Sir Martin Evans Building, Museum Avenue, Cardiff CF10 3AX, UK; alalawis@cardiff.ac.uk (S.A.); rifqid@cardiff.ac.uk (D.R.); alahmadiag@cardiff.ac.uk (A.A.); alotibirm@cardiff.ac.uk (R.A.); alradif@cardiff.ac.uk (F.A.); alshehrin@cardiff.ac.uk (N.A.); yhc42@cam.ac.uk (Y.-H.C.); chenJ91@cardiff.ac.uk (J.C.); albalawife@cardiff.ac.uk (F.A.); guschinaia@cardiff.ac.uk (I.A.G.); 2 Ministry of Health, P.O. Box 393, Muscat 100, Oman; 3 European Cancer Stem Cell Research Institute, Cardiff School of Biosciences, Cardiff University, Hadyn Ellis Building, Maindy Road, Cardiff CF24 4HQ, UK; staha@kfmc.med.sa; 4 Medical Laboratory Sciences, Health Sciences, Oman College of Health Sciences, P.O. Box 3720, Muscat 112, Oman; nabras.almahrami@ochs.edu.om; 5 Division of Infection and Immunity, School of Medicine, Cardiff University, Henry Wellcome Building, Heath Park, Cardiff CF14 4XN, UK; hughestr@cardiff.ac.uk

**Keywords:** atherosclerosis, gene expression, inflammation, macrophages, nutraceuticals, plaque stability, punicalagin, urolithins

## Abstract

Nutraceuticals are emerging as promising agents for the prevention and treatment of atherosclerosis, particularly in light of the limitations associated with current pharmacotherapies. Pomegranate-derived polyphenols, especially punicalagin (PC), possess multiple cardioprotective properties. However, their direct biological effects are constrained by poor absorption and low bioavailability. Instead, many of their actions are mediated by gut microbiota-derived metabolites known as urolithins. Despite this, the roles of PC and its metabolites in atherosclerosis remain inadequately defined. The objective of this study was to investigate the anti-atherogenic effects and underlying mechanisms of PC and its major metabolites—ellagic acid and urolithins A, B, C, and D—using in vitro and in vivo approaches. In vitro, these compounds broadly inhibited key pro-atherogenic processes in macrophages and endothelial cells, including reactive oxygen species production and inflammatory gene expression, with notable metabolite-specific differences. Urolithin A (UA), identified as the most effective compound, was further evaluated in LDL receptor-deficient mice fed a high-fat diet. UA supplementation improved peripheral blood immune cell profile, reduced atherosclerotic plaque burden and inflammation, and enhanced markers of plaque stability. RNA sequencing of the thoracic aorta revealed key molecular pathways underlying the protective actions of UA. Collectively, these findings highlight the therapeutic potential of PC-derived metabolites, particularly UA, in combating atherosclerosis and support the need for future human clinical studies.

## 1. Introduction

Atherosclerotic cardiovascular disease (ASCVD), a chronic inflammatory disorder of the vasculature, is a major cause of morbidity and mortality worldwide and represents a substantial healthcare and economic burden [1]. ASCVD is triggered by various risk factors, particularly elevated plasma levels of low-density lipoprotein (LDL) cholesterol, that lead to the progression of the disease during the lifespan of an individual, which includes endothelial cell dysfunction; recruitment of immune cells such as monocytes, their differentiation into macrophages and subsequent transformation into foam cells via uptake of modified LDL; formation of lipid-rich necrotic core from death of foam cells that then exacerbates inflammation; migration of smooth muscle cells (SMC) from media to the intima where they stabilise plaques via the production of extracellular matrix (ECM) of the fibrous plaque; and plaque rupture because of thinning of the fibrous cap via increased actions of proteases under inflammatory conditions leading to thrombosis and clinical complications such as myocardial infarction and cerebrovascular accidents [1].

Current pharmacotherapies against ASCVD are associated with various issues such as high residual risk for the disease, various side effects, and high cost associated with some (e.g., neutralising monoclonal antibodies) [1]. Nutraceuticals, food components with health benefits beyond their nutritional value, represent promising agents against ASCVD, but our understanding of the molecular mechanisms underlying their protective actions remains relatively poor [1]. Ellagitannins (ET) are polyphenols present at high concentrations in many fruits and nuts, such as pomegranate, raspberries, walnuts, and almonds, all of which have demonstrated health benefits [2]. The most abundant ET polyphenols are punicalagin (PC) and ellagic acid (EA); however, these are poorly absorbed by the intestine because they are very large and hydrophobic molecules [2]. PC from pomegranates and other sources is mainly hydrolysed to EA in the acidic environment of the stomach [2]. EA then undergoes a series of metabolic transformations by the gut microbiota to produce urolithins, with urolithin (U)A and UB being the two key final products [2] (see Supplementary Figure S1 for structures of PC, EA, and various urolithins).

Isolated studies have demonstrated cardioprotective actions of PC, EA, or urolithins in vitro, in animal model systems, and in humans [2,3,4,5]. Such beneficial effects include inhibition of oxidised LDL (oxLDL) levels, oxLDL-mediated responses, cholesterol biosynthesis, plasma lipid levels, and inflammatory marker production, together with stimulation of cholesterol efflux and endothelial cell function [2,3,4,5,6,7]. However, there are many limitations associated with these studies. For example, human trials in relation to ASCVD and other vascular metabolic diseases have produced mixed results, which possibly reflects the very low number of participants along with differences in study design, concentration of the agent, and duration of the intervention [2]. In addition, only a few studies on animal models of ASCVD have been carried out, and these have been restricted to the Apolipoprotein-E (ApoE)-deficient mouse model, where, in most cases, only limited parameters have been analysed [4,5,8]. However, the lipoprotein profile in these mice is different from that seen in humans, and, additionally, this is perceived as a more aggressive mouse model for atherosclerosis, given that the ApoE protein impacts inflammation, haematopoietic stem cell proliferation, and monocytosis [9]. On the other hand, the LDL receptor-deficient mice (LDLr^−/−^) do not have many such limitations and also have a more human-like plasma lipid profile characteristic of familial hypercholesterolemia [9].

To our knowledge, no studies have compared the effects of PC, EA, and different urolithins together on key cellular processes associated with atherosclerosis in vitro. The objective of this study was hence to investigate the actions of PC, EA, UA, UB, UC, and UD on monocytes/macrophages and endothelial cells in vitro and to extend the investigation of the actions of UA, which was the most effective agent, to the LDLr^−/−^ model system in vivo. RNA-sequencing (RNA-seq) of the thoracic aorta and subsequent bioinformatic analyses were also employed to further probe the underlying molecular mechanisms. Our studies provide new insights into the protective actions of PC and its metabolites in the regulation of several atherosclerosis-associated processes in vitro, together with the ability of UA to improve peripheral blood immune cell profile, attenuate plaque burden and inflammation, promote plaque stability, and regulate key pathways implicated in the disease.

## 2. Materials and Methods

### 2.1. Materials

PC, EA, UB, human monocytic THP-1 cell line and human aortic endothelial cells (HAEC) were purchased from Sigma-Aldrich (Gillingham, UK), UC and UD were from Insight Biotechnology Limited (Welwyn Garden City, UK), UA was from NewChem Technologies Limited (Newcastle upon Tyne, UK) or Sigma-Aldrich (Gillingham, UK); lactate dehydrogenase (LDH) assay kit (C20301), Dil-oxLDL and optimum cutting temperature (OCT) embedding matrix were from Thermo Fisher Scientific (Altrincham, UK); Lymphoprep^TM^ was from STEMCELL Technologies (Cambridge, UK); and tumour necrosis factor-α (TNF-α), interferon-γ (IFN-γ) and monocyte chemotactic protein-1 (MCP-1) were from Peprotech (London, UK). All the other reagents were from Sigma-Aldrich (Gillingham, UK) unless otherwise stated.

### 2.2. Cell Culture and In Vitro Assays

HAEC were cultured in their respective ready-to-use media as per manufacturer’s instructions (Sigma-Adrich, Gillingham, UK). Culturing of human THP-1 monocytes, macrophages obtained from them following their differentiation using 0.16 μM phorbol 12-myristate 13-acetate (PMA) for 24 h, and human monocyte-derived macrophages (HMDM) obtained from monocytes of buffy coats was performed as in our previous studies [10,11,12,13,14]. PC and its metabolites for these in vitro experiments were dissolved in dimethyl sulfoxide (DMSO), which therefore served as a vehicle control.

Determination of cell viability using the LDH Cytotoxicity Assay Kit (C20301, Thermo Fisher Scientific, Altrincham, UK), MCP-1-driven monocytic migration using modified Boyden chambers (Thermo Fisher Scientific, Altricham, UK), production of reactive oxygen species (ROS) using the 2′,7′-dichorofluorescin diacetate (DCFDA) cellular ROS detection kit (ab113851, Abcam, Cambridge, UK), macropinocytosis, Dil-oxLDL uptake and cholesterol-crystal-mediated production of interleukin (IL)-1β using an ELISA kit (DLB50, R&D Systems, Abingdon, UK) were carried out as our previous studies [10,11,12,13,14,15]. In the assay for monitoring ROS production, the cells were first stained with DCFDA and, following its removal, incubated with tert-butyl hydroperoxide (TBHP) in the presence of the vehicle or PC and its metabolites (cells incubated with the vehicle in the absence of TBHP were also included for comparison) (ab113851, Abcam, Cambridge, UK). Total RNA was prepared from cells using RiboZol^TM^ (Avantor, Lutterworth, UK) and real-time quantitative polymerase chain reaction (RT-qPCR) with primers against MCP-1 (5′-CGCTCAGCCAGATGCAATCAATG-3′ and 5′-ATGGTCTTGAAGATCACAGCTTCTTTGG-3′), intercellular adhesion molecule-1 (ICAM1) (5′-GACCAGAGGTTGAACCCCAC-3′ and 5′-GCGCCGGAAAGCTGTAGAT-3′) and glyceraldehyde 3-phosphate dehydrogenase (GAPDH) gene (5′-CTTTTGCGTCGCCAGCCGAG-3′ and (5′-GCCCAATACGACCAAATCCGTTGACT-3′) and data analysis via the ΔΔct method was performed as per our previous studies [10,11,12,13,14].

### 2.3. Animal Experiments

These were carried out according to the Guide for Care and Use of Laboratory Animals (NIH Publication No. 85-23; revised 1996) and approved by Cardiff University’s Ethics Review Committee and the United Kingdom Home Office (licence 30/3365 and P5211628) [10,11,13,14,15]. The LDLr^−/−^ mice, homozygous for the LDLrtm1Her mutation and backcrossed to the C57BL/6J strain (Jackson Laboratory, Bar Harbor, ME, USA), were expanded locally in a pathogen-free and light- and temperature-controlled facility (lights on 7 a.m. to 7 p.m., 22 °C) [10,11,13,14,15]. Male LDLr^−/−^ mice (8-week-old) were randomly assigned to two groups and fed a high-fat diet (HFD) [21% (*w*/*w*) pork lard and 0.15% (*w*/*w*) cholesterol] alone or supplemented with 50 mg/kg/day of UA for 12 weeks. The concentration of UA, which is 4.05 mg/kg/day human equivalent based on the guide for dose conversion between animals and humans [16], was based on previous studies [17,18,19,20]. The 12-week duration of intervention was similar to our other in vivo studies on nutraceuticals [10,11,14].

The weight of the animals at the start of the study and frequently during the feeding period (2 days/week), together with the weight of the supplied food and that remaining, was recorded. The levels of circulating myeloid and lymphoid cells in the peripheral blood were determined a day before sacrifice, as in our previous studies [14]. The mice were sacrificed using CO_2_ asphyxiation (death confirmed via absence of a pulse), and various tissues were weighed, snap frozen, and stored at −80 °C. Blood following cardiac puncture was collected in the presence of 50 U/mL heparin, and plasma obtained following centrifugation (10 min at 12,000× *g*). For cryosectioning, the heart was perfused with phosphate-buffered saline (PBS), mounted with OCT embedding matrix, and flash frozen [10,11,13,14].

### 2.4. Lipid Analysis

The plasma concentrations of LDL/very low-density lipoprotein (VLDL) cholesterol (LDL/VLDL-C), high-density lipoprotein (HDL) cholesterol (HDL-C), cholesteryl esters (CE) and total cholesterol (TC) were measured using the Cholesterol Assay Kit-HDL and LDL/VLDL (ab65390, Abcam, Cambridge, UK) whereas that of triacylglycerol (TG) was determined using the Triglyceride Assay Kit (ab65336, Abcam, Cambridge, UK) as our previous studies [10,11,13,14,15].

Short-chain fatty acids (SCFAs) in the plasma and the faeces were extracted using a solution of orthophosphoric acid and analysed by capillary gas chromatography (GC). All procedures, including centrifugations, were carried out at 4 °C. Thus, 20–30 mg of faecal samples were thawed, weighed, and homogenised for 3 min in 100 mL of 16% (*v*/*v*) aqueous orthophosphoric acid, whereas the plasma samples (10–20 µL) were acidified with 2 µL of 70% (*v*/*v*) orthophosphoric acid. A known amount of 2-ethylbutyric acid (Sigma-Aldrich, Gillingham, UK) was added as an internal standard to aid the subsequent quantification of fatty acids. The suspensions or plasma samples were kept at room temperature for 10 min with occasional vortexing and then centrifuged at 13,000× *g* for 20 min. GC was then performed using a Clarus 500 gas chromatograph with a flame ionising detector (PerkinElmer 8500, PerkinElmer, Waltham, MA, USA) fitted with a TR-FFAP 30 m × 0.32 mm i.d. × 0.25 μm capillary column (Thermo Fisher Scientific, Altricham, UK). Two temperature operation conditions were used for effective separation of individual compounds: (1) 100 °C for 3 min, programmed to 220 °C at 4 °C/min, hold for 13 min; and (2) 90 °C for 1 min, programmed to 130 °C at 10 °C/min, hold for 3 min, and then programmed to 200 °C at 10 °C/min followed by hold for 8 min. Nitrogen was used as a carrier; the injector temperature was 220 °C, the detector temperature was 240 °C, and 5 μL was the injection volume. SCFAs were identified routinely by comparing retention times of peaks with those of standards: acetic acid (Thermo Fisher Scientific, Altricham, UK) and propionic, valeric, and butyric acids (Sigma-Aldrich, Gillingham, UK). Total Chrom Navigator software, version 6.2.1 (PerkinElmer, Waltham, MA, USA) was used for data acquisition.

### 2.5. Plaque Analyses

Histological and immunohistological staining of aortic root sections (8 μm) was carried out as in our previous studies [10,11,13,14]. Image analysis was carried out in a blinded fashion using the ImageJ software (2.9.0/v1.54b), as in our previous studies [10,11,13,14].

### 2.6. RNA-Sequencing (RNA-Seq)

The thoracic aorta was stored in RNAlater^TM^ stabilisation solution (Thermo Fisher Scientific, Altrincham, UK) at −80 °C, and total RNA was isolated using TissueLyser II containing one 2 mm stainless steel bead and the RNeasy Mini Kit as described by the manufacturer (Qiagen, Manchester, UK). High-quality RNA (integrity number typically >6.8) was then subjected to RNA-seq and standard bioinformatic analysis at Novogene (Cambridge, UK). This included quality check, preparation of mRNA library (poly A enrichment), Illumina sequencing PE150 with data quality control and data filtering, mapping to reference genome, quantification of gene expression and correlation analysis, differential expression and enrichment analyses such as Gene Ontology (GO) and Kyoto Encyclopedia of Genes and Genomes (KEGG) pathway of differentially expressed genes (DEGs) [14]. Additional DEG analyses (*p* adjusted < 0.05 filter), such as canonical pathways, diseases, and function, were performed via Ingenuity Pathway Analysis (IPA) software (https://www.qiagen.com/ja-us/products/discovery-and-translational-research/next-generation-sequencing/informatics-and-data/interpretation-content-databases/ingenuity-pathway-analysis (accessed on 15 November 2023)) (Qiagen, Manchester, UK) [14]. The data have been submitted to the GEO repository (GSE311266).

### 2.7. Statistical Analyses of Data

The normality of data was evaluated via a Shapiro–Wilk test, and statistical analysis of data from two groups only was carried out using an unpaired Student’s *t*-test for data that were normally distributed or a Mann–Whitney U test if this was not the case. A one-way analysis of variance (ANOVA) followed by either Tukey’s (for equal variances) or Dunnett’s or Dunnett’s T3 (for unequal variances) post hoc test was employed for normally distributed data involving more than two groups, or via Kruskal–Wallis and Dunn’s post hoc test if the data were not normally distributed. GraphPad Prism 9 software was used for statistical analysis, with significance defined by *p* ≤ 0.05.

## 3. Results

### 3.1. Dose Response Experiments on PC and Its Metabolites on ROS Production and Macropinocytosis In Vitro

The actions of PC and its metabolites on macrophages were first investigated, given their important roles at all stages of the disease [1]. The studies initially employed several different concentrations of PC and its metabolites (25 μM, 50 μM, 75 μM, 100 μM, and 150 μM) on the THP-1 cell line, which is extensively used for studies on monocytes and macrophages in ASCVD, with conservation of responses with primary cultures and in vivo [10,11,12,14,21]. All these concentrations of PC and its metabolites had no effect on the viability of THP-1 macrophages (Supplementary Figure S2).

**Figure 1:**
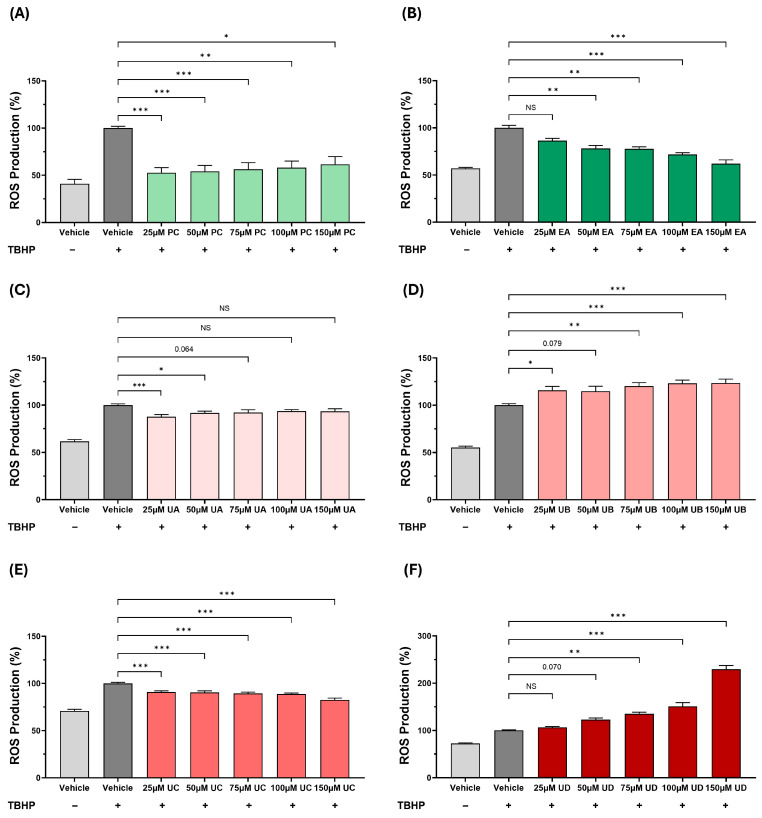
The effects of PC and its metabolites on the TBHP-induced ROS production in THP-1 macrophages. The cells were treated with TBHP and either the DMSO vehicle (vehicle control) or the indicated concentration of PC or its metabolites. Cells treated with the vehicle in the absence of TBHP were included for comparison (no TBHP control). ROS production was measured using the DCFDA Cellular ROS Detection Assay kit (ab113851, Abcam, Cambridge, UK). Data (mean ± SEM from five independent experiments) are presented as a percentage of the vehicle control (arbitrarily set to 100%). Statistical analysis was carried out using a one-way ANOVA with Dunnett post hoc test (**C**,**E**) or Kruskal–Wallis with Dunn’s post hoc test (**A**,**B**,**D**,**F**) (*, *p* ≤ 0.05; **, *p* ≤ 0.01; ***, *p* ≤ 0.001; NS, not significant or as indicated).

The production of ROS is an important early event in atherosclerosis, being responsible for the oxidation of LDL [1]. We therefore analysed the effects of PC and its metabolites on the TBHP-induced ROS production (Figure 1). This TBHP-induced ROS production was significantly attenuated by PC at all concentrations (*p* ≤ 0.001 at 25 μM, 50 μM and 75 μM, *p* = 0.004 at 100 μM and *p* = 0.029 at 150 μM) (Figure 1A), by EA at 50 μM (*p* = 0.007), 75 μM (*p* = 0.005), 100 μM (*p* ≤ 0.001) and 150 μM (*p* ≤ 0.001) (Figure 1B), by UA at 25 μM (*p* ≤ 0.001) and 50 μM (*p* = 0.045) with a trend towards reduction at 75 μM (*p* = 0.064) (Figure 1C), and by UC at all concentrations (*p* ≤ 0.001 in all cases) (Figure 1E). In contrast, the TBHP-induced ROS production was increased by UB at 25 μM (*p* = 0.032), 75 μM (*p* = 0.003), 100 μM (*p* ≤ 0.001) and 150 μM (*p* ≤ 0.001) with a trend towards increase at 50 μM (*p* = 0.079) (Figure 1D), and by UD at 75 μM (*p* = 0.002), 100 μM (*p* ≤ 0.001) and 150 μM (*p* ≤ 0.001) with a trend towards increase at 50 μM (*p* = 0.070) (Figure 1F).

**Figure 2:**
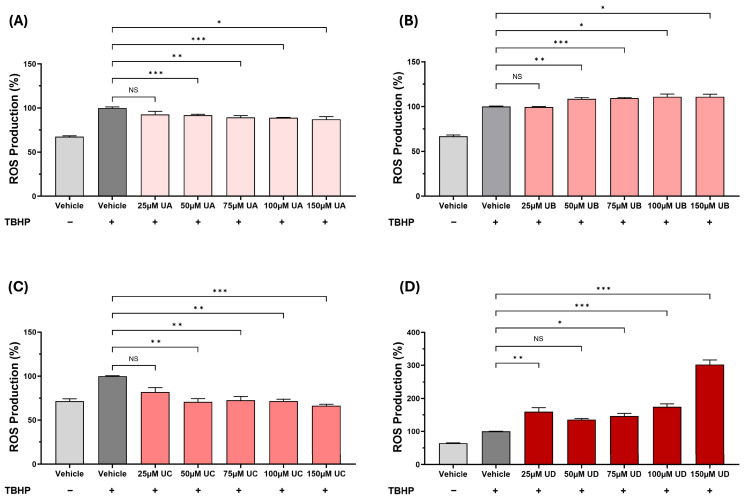
The effects of urolithins on the TBHP-induced ROS production in THP-1 monocytes. THP-1 monocytes were treated with TBHP and either the DMSO vehicle (vehicle control) or the indicated concentration of UA, UB, UC, and UD. Cells incubated with the vehicle in the absence of TBHP were included for comparison (no TBHP control). ROS production is displayed as a percentage of the vehicle control (arbitrarily set to 100%). Data are presented as mean ± SEM from five independent experiments. Statistical analysis was performed using a one-way ANOVA with Dunnett T3 post hoc test (**A**,**B**) or Kruskal–Wallis with Dunn’s post hoc test (**C**,**D**), where * *p* ≤ 0.05, ** *p* ≤ 0.01, *** *p* ≤ 0.001, and NS, not significant.

The studies on THP-1 macrophages showed that from the urolithins, UA and UC had antioxidant activities, whereas UB and UD had pro-oxidant actions. Further experiments with these four urolithins also showed that such an action extended to THP-1 monocytes. Thus, UA and UC inhibited the TBHP-induced ROS production at 50 μM (*p* ≤ 0.001 and *p* = 0.003, respectively), 75 μM (*p* = 0.003 and *p* = 0.002, respectively), 100 μM (*p* ≤ 0.001 and *p* = 0.005, respectively), and 150 μM (*p* = 0.015 and *p* ≤ 0.001, respectively) (Figure 2A,C). In contrast, UB increased the TBHP-induced ROS production at 50 μM (*p* = 0.003), 75 μM (*p* ≤ 0.001), 100 μM (*p* = 0.036), and 150 μM (*p* = 0.024) (Figure 2B), and UD significantly increased this at 25 μM (*p* = 0.002), 75 μM (*p* = 0.049), 100 μM (*p* ≤ 0.001), and 150 μM (*p* ≤ 0.001) (Figure 2D). Therefore, further studies on urolithins focused on UA and UC because of their antioxidant actions in both THP-1 monocytes and macrophages.

**Figure 3:**
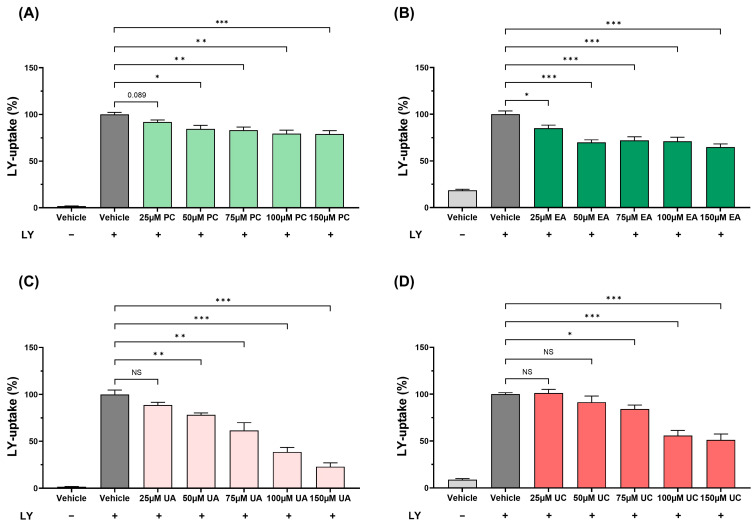
PC and its metabolites significantly inhibit macropinocytosis in THP-1 macrophages. THP-1 macrophages were treated with different concentrations of PC, EA, UA, and UC (25–150 µM) or the DMSO vehicle control and 100 µg/mL LY (+) for 24 h (cells incubated with vehicle alone without LY were also included for comparison). The uptake of LY was monitored by flow cytometry. The results are presented as percentages (mean ± SEM) of the vehicle control (assigned 100%) from three independent experiments. Statistical analysis was performed using a one-way ANOVA with Dunnett T3 (**A**,**C**,**D**) or Dunnett (**B**) post hoc test (*, *p* ≤ 0.05; **, *p* ≤ 0.01; ***, *p* ≤ 0.001; NS, not significant or as indicated).

Macropinocytosis is increasingly being found to play an important role in atherosclerosis via the intake of lipids and lipoproteins, and can be followed by monitoring the uptake of the Lucifer Yellow (LY) dye [11,12,13]. The effects of different concentrations of PC, EA, UA, and UC on LY uptake by THP-1 macrophages were therefore investigated. LY uptake was attenuated by PC at 50 μM (*p* = 0.021), 75 μM (*p* = 0.004), 100 μM (*p* = 0.002) and 75 μM (*p* = 0.001) with a trend towards reduction at 25 μM (*p* = 0.089), by EA at all concentrations (*p* = 0.028 at 25 μM and *p* ≤ 0.001 at all other concentrations), by UA at 50 μM (*p* = 0.008), 75 μM (*p* = 0.007), 100 μM (*p* ≤ 0.001) and 150 μM (*p* ≤ 0.001) and by UC at 75 μM (*p* = 0.033), 100 μM (*p* ≤ 0.001) and 150 μM (*p* ≤ 0.001) (Figure 3).

The concentration of PC and its metabolites for subsequent experiments was selected based on the outcomes of the assays detailed above: 50 µM PC (lowest concentration when a significant reduction in LY uptake was seen; trend towards reduction at 25 µM) (Figure 3A); 50 µM EA (lowest concentration when a significant decrease in the TBHP-induced ROS production in THP-1 macrophages was seen; not significant at 25 µM) (Figure 1B); 50 µM UA [lowest concentration when a significant reduction in both the TBHP-induced ROS production in THP-1 monocytes (Figure 2A) and LY uptake (Figure 3C) was observed; not significant at 25 µM); and 100 µM UC [lowest concentration when a marked significant inhibition of LY uptake was observed (*p* ≤ 0.001; not significant at 50 µM and *p*-value of only 0.033 at 75 µM) (Figure 3D)].

### 3.2. The Effects of PC and Its Metabolites on ROS Production in Primary Cultures of Human Monocyte-Derived Macrophages and Human Aortic Endothelial Cells

**Figure 4:**
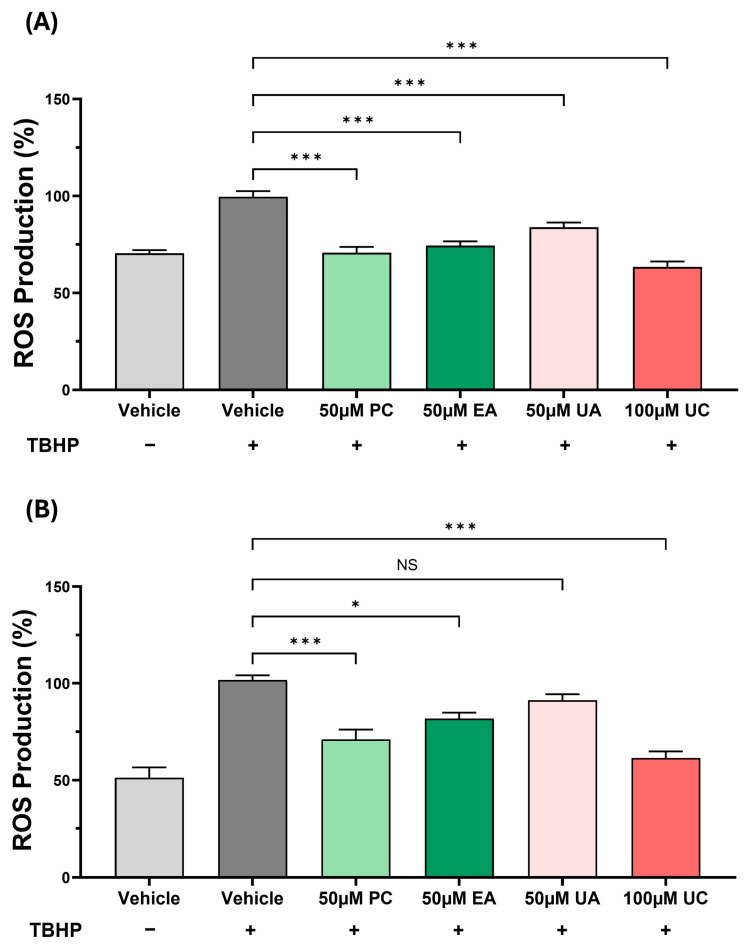
The effects of PC and its metabolites on the TBHP-induced ROS production in HMDM and HAEC. HMDM (**A**) or HAEC (**B**) were treated with TBHP and either the DMSO vehicle (control) or the indicated concentration of PC and its metabolites. Cells treated with vehicle in the absence of TBHP were also included for comparison. ROS production is presented as a percentage of the vehicle control seen in the presence of TBHP (arbitrarily set to 100%). Statistical analyses of data (mean ± SEM from four independent experiments) were performed using one-way ANOVA with Dunnett post hoc test (**A**) or Kruskal–Wallis with Dunn’s post hoc test (**B**) (*, *p* ≤ 0.05; ***, *p* ≤ 0.001; NS, not significant).

The effects of PC and its metabolites on the TBHP-induced ROS production were currently restricted to THP-1 monocytes/macrophages (Figure 1 and Figure 2). To rule out whether this was peculiar to the use of the cell line and to extend the findings to other key cell types associated with atherosclerosis, the assay was repeated in primary cultures of HMDM and HAEC. In HMDM, the TBHP-induced ROS production was significantly attenuated by PC, EA, UA, and UC (*p* ≤ 0.001 in all cases) (Figure 4A). For HAEC, the TBHP-induced ROS production was significantly reduced by PC (*p* ≤ 0.001), EA (*p* = 0.016), and UC (*p* ≤ 0.001), whereas the decrease by UA was not significant (Figure 4B).

### 3.3. The Effects of PC and Its Metabolites on Chemokine-Induced Monocytic Migration and oxLDL Uptake

**Figure 5:**
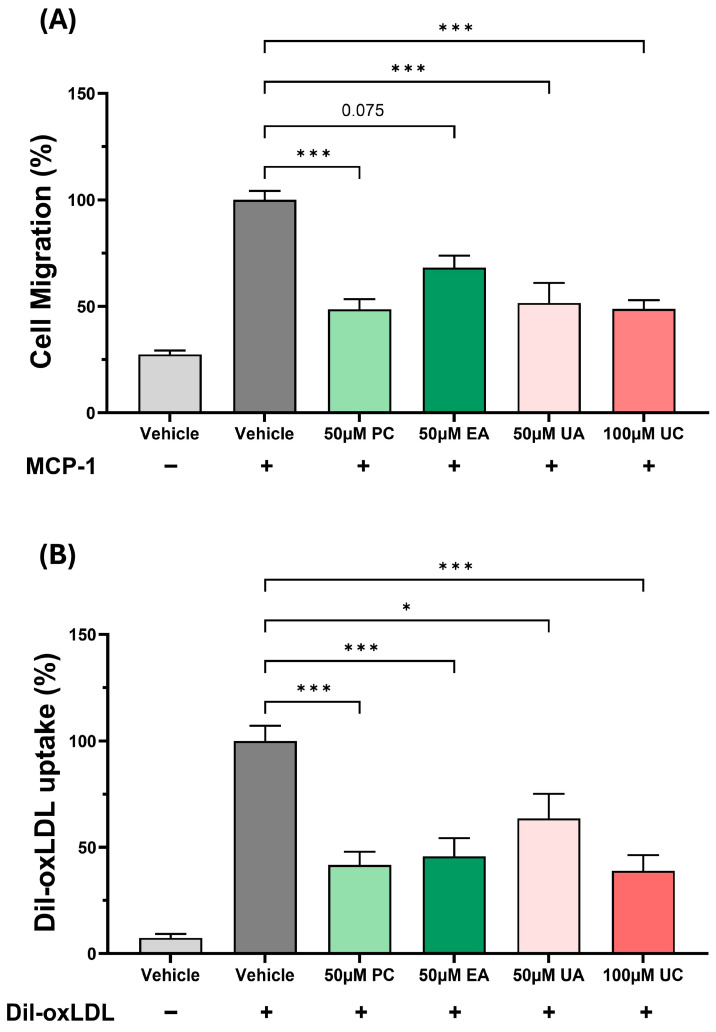
The effects of PC and its metabolites on chemokine-induced monocytic migration and oxLDL uptake. For chemokine-induced monocytic migration (**A**), THP-1 monocytes were incubated for 3 h with the DMSO vehicle in the absence or the presence of MCP-1 (20 ng/mL), or with MCP-1 in the presence of PC and its metabolites. The migration of monocytes was then determined with that from vehicle-treated cells in the presence of MCP-1, arbitrarily assigned as 100%. For oxLDL uptake (**B**), HMDM were incubated with the DMSO vehicle or the indicated concentrations of PC and its metabolites for 1 h prior to the addition of 5 μg/mL Dil-oxLDL for a further 24 h. Cells incubated with the vehicle in the absence of Dil-oxLDL were also included for comparison. Dil-oxLDL uptake was monitored by flow cytometry. The results are mean ± SEM from three independent experiments with statistical analysis carried out using Kruskal–Wallis with Dunn’s post hoc test (**A**) or one-way ANOVA followed by Dunnett post hoc test (**B**) (* *p* ≤ 0.05; *** *p* ≤ 0.001; or as indicated).

The chemokine-driven monocytic migration represents a key early event in the pathogenesis of atherosclerosis and subsequent foam cell formation [1]. The effects of PC and its metabolites on monocytic migration triggered by the key chemokine MCP-1 were therefore investigated. As shown in Figure 5A, the MCP-driven monocyte migration was significantly inhibited by PC, UA, and UC (*p* ≤ 0.001 in all cases), whereas a trend towards reduction was seen with EA (*p* = 0.075) (Figure 5A).

The macrophage uptake of modified LDL is a critical early event in atherogenesis [1] and hence this was investigated in HMDM using Dil-oxLDL. As shown in Figure 5B, the Dil-oxLDL uptake was inhibited significantly when the cells were treated with PC, EA, UA, and UC (*p* ≤ 0.001 for PC, EA, and UC and *p* = 0.018 for UA).

### 3.4. Anti-Inflammatory Actions of PC and Its Metabolites

**Figure 6:**
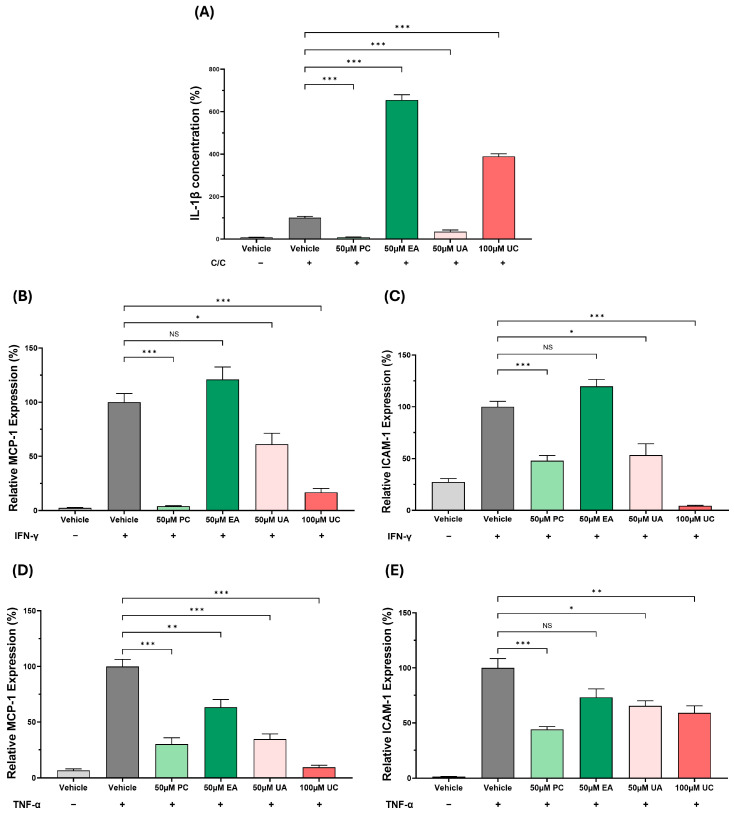
The effects of PC and its metabolites on pro-inflammatory responses in macrophages and endothelial cells. For IL-1β secretion (**A**), THP-1 macrophages were stimulated with cholesterol crystals (C/C) and treated with the DMSO vehicle or the indicated concentration of PC or its metabolites. Cells treated with vehicle alone in the absence of cholesterol crystals were included for comparative purposes. IL-1β concentration was determined by ELISA and expressed as a percentage of the vehicle control, which was arbitrarily set to 100. For cytokine-induced gene expression, THP-1 macrophages (**B**,**C**) or HAEC (**D**,**E**) were treated for 3 h with IFN-γ (**B**,**C**) or TNF-α (**D**,**E**) followed by the vehicle or the indicated concentration of PC or its metabolites for 24 h. Cells incubated with the vehicle in the absence of the cytokine were also included for comparison. RT-qPCR was used to measure the expression of MCP-1 (**B**,**D**) or ICAM-1 (**C**,**E**). The levels of mRNA expression were compared using the ΔΔCT method and normalised to the GAPDH housekeeping gene. The normalised gene expression in cytokine-treated cells in the presence of the vehicle has been arbitrarily assigned as 100%. Data (mean ± SEM) are from three (**A**) or four (**B**–**E**) independent experiments with statistical analysis performed using a one-way ANOVA with Dunnett T3 post hoc test (*, *p* ≤ 0.05; **, *p* ≤ 0.01; ***, *p* ≤ 0.001; NS, not significant).

Cholesterol crystals induce IL-1β secretion in THP-1 macrophages produced by PMA stimulation predominantly via the inflammasome pathway [14]. The effects of PC and its metabolites on IL-1β secretion were therefore analysed. As shown in Figure 6A, the IL-β levels were reduced significantly in the presence of PC and UA (*p* ≤ 0.001 in both cases). In contrast, the IL-1β levels were significantly increased by EA or UC (*p* ≤ 0.001 in both cases).

The cytokine IFN-γ plays a key role in promoting atherosclerosis, in part by inducing the expression of pro-inflammatory genes such as MCP-1 and ICAM-1 in macrophages [14]. The effects of PC and its metabolites on such IFN-γ responses were therefore investigated in THP-1 macrophages. For MCP-1, the IFN-γ-induced expression was significantly attenuated by PC (*p* ≤ 0.001), UA (*p* = 0.037), and UC (*p* ≤ 0.001), whereas EA had no significant effect (Figure 6B). For ICAM-1, the IFN-γ-induced expression was attenuated by PC (*p* ≤ 0.001), UA (*p* = 0.013), and UC (*p* ≤ 0.001), whereas EA had no significant effect (Figure 6C).

To investigate whether the anti-inflammatory actions of PC and its metabolites extended beyond macrophages, their effects on the TNF-α-induced MCP-1 and ICAM-1 expression associated with endothelial cell dysfunction were analysed using HAEC. The TNF-α-induced MCP-1 expression was significantly decreased by PC, EA, UA, and UC (*p* = 0.006 for EA and *p* ≤ 0.001 for the others). For the TNF-α-induced ICAM expression, this was significantly attenuated by PC (*p* ≤ 0.001), UA (*p* = 0.016), and UC (*p* = 0.008), whereas EA had no significant effect (Figure 6E).

The studies detailed above showed that PC, EA, UA, and UC had many anti-atherogenic actions in vitro, thereby warranting further studies in vivo. These were carried out on UA because of the poorly documented bioavailability of PC and EA, and as UA is a final product of PC metabolism [2] with many potent inhibitory actions against pro-atherogenic changes (i.e., inhibition of TBHP-induced ROS production in monocytes and macrophages, chemokine-driven monocytic migration, Dil-oxLDL uptake and macropinocytosis, and pro-inflammatory gene expression in both macrophages and endothelial cells).

### 3.5. UA Decreases Plaque Burden and Inflammation and Produces a Stable Plaque Phenotype

**Figure 7:**
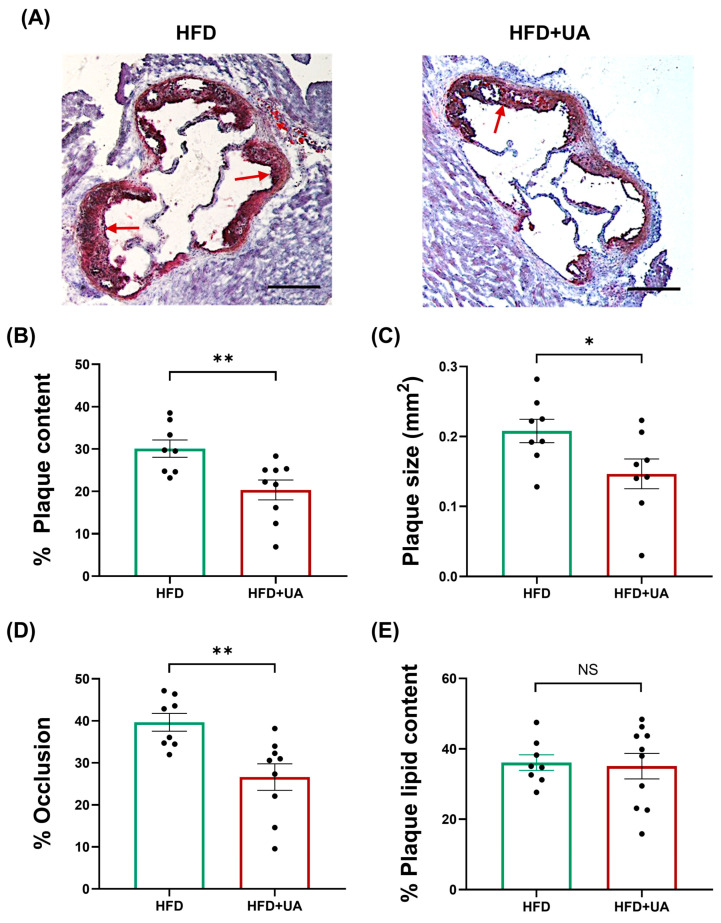
UA attenuates plaque burden in HFD-fed LDLr^−/−^ mice. LDLr^−/−^ mice were given either an HFD or an HFD supplemented with UA (HFD+UA) for 12 weeks. Aortic root sections were stained with oil red O (ORO), images captured using a Leica DMRB microscope (Leica Microsystems, Milton Keynes, UK) under ×5 magnification, and then analysed using the ImageJ software. Representative images are shown in panel (**A**) (scale bar of 400 μm; arrows indicate ORO staining in plaques). The graphs show plaque content calculated as the percentage of plaque area within the total vessel area (**B**), plaque size (**C**), occlusion measured as the percentage of plaque area within the lumen area (**D**), and plaque lipid content (**E**). Data are mean ± SEM [*n* = 8 for HFD in (**B**–**E**) and HFD+UA in (**C**); *n* = 9 for HFD+UA in (**B**,**D**); *n* = 10 for HFD+UA for (**E**)] with statistical analysis performed using an unpaired Student’s *t*-test (*, *p* ≤ 0.05; **, *p* ≤ 0.01; NS, not significant).

**Figure 8:**
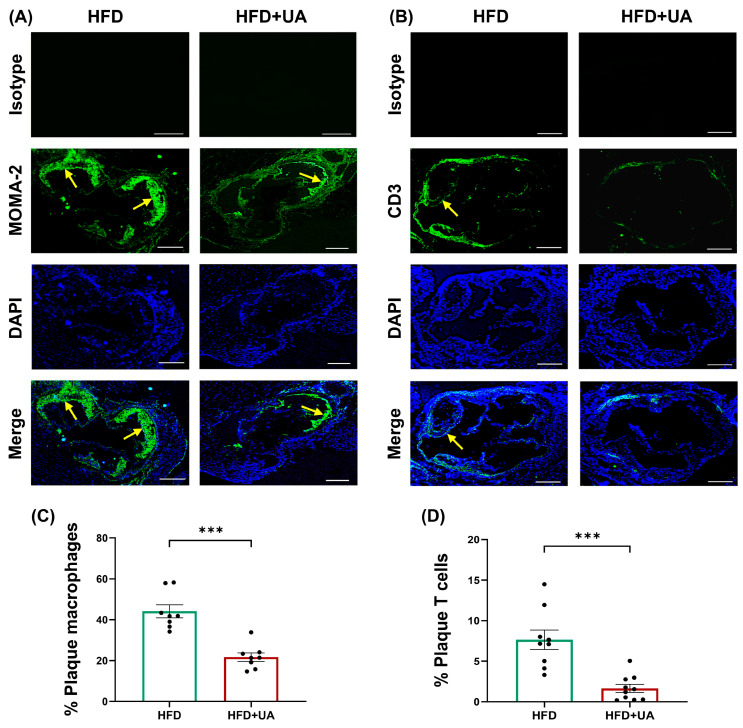
UA produces a significant reduction in plaque macrophage and CD3 + T cell content in HFD-fed LDLr^−/−^ mice. LDLr^−/−^ mice were given either an HFD or an HFD supplemented with UA (HFD+UA) for 12 weeks. Sections of the aortic root were subjected to immunofluorescence staining to detect the presence of MOMA-2+ macrophages (**A**,**C**) or CD3+ T cells (**B**,**D**). The images were acquired with an Olympus BX61 microscope (Evident Scientific, Stansted, UK) (×4 magnification) with quantification performed using ImageJ software. A representative image (scale bar 400 μm; arrows indicate macrophage/CD3+ T cell staining in plaques) is shown in panels A and B with graphs displaying the percentage of MOMA-2+ macrophages (**C**) or CD3+ T cells (**D**) in the plaque. Data are mean ± SEM (*n* = 8 for HFD and HFD+UA in (**C**); *n* = 9 for HFD in (**D**); and *n* = 10 for HFD+UA in (**D**)) with statistical analysis performed using an unpaired Student’s *t*-test (***, *p* ≤ 0.001).

**Figure 9:**
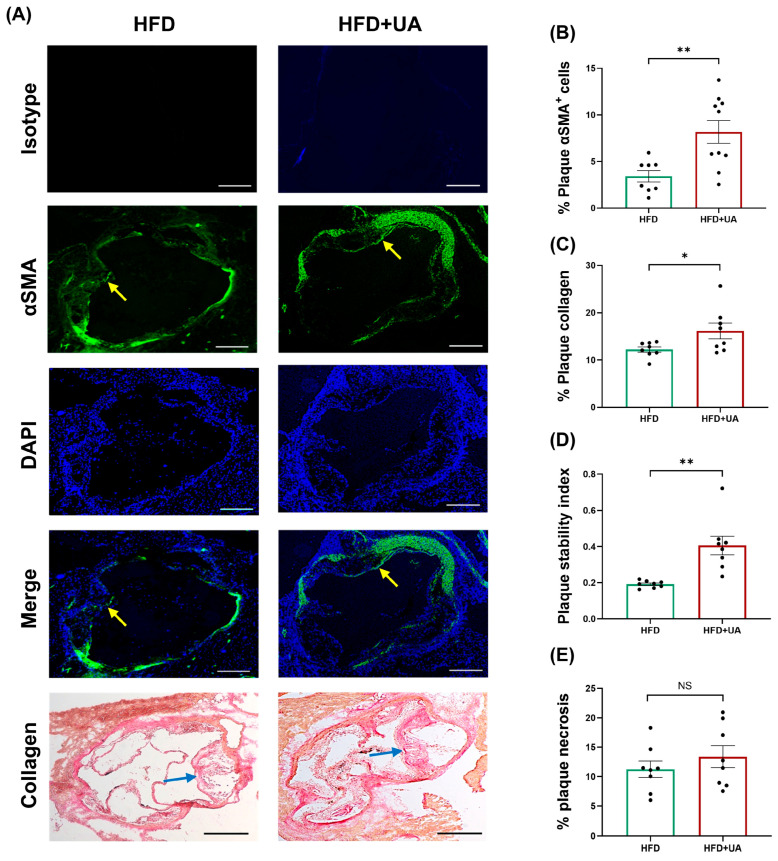
UA produces a stable plaque phenotype in HFD-fed LDLr^−/−^ mice. LDLr^−/−^ mice were fed either HFD or HFD supplemented with UA (HFD+UA) for 12 weeks, and sections from the aortic root were subjected to immunofluorescence staining to detect SMC or Van Gieson’s staining to determine collagen. Images were captured using the Olympus BX61 microscope (Evident Scientific, Stansted, UK) for SMC (×4 magnification) and Leica DMRB microscope for collagen (Leica Microsystems, Milton Keynes, UK) (×5 magnification) and analysed using the ImageJ software. Representative images with a scale bar of 400 μm are shown in (**A**) (arrows indicate *α*SMA+/collagen staining in plaques), with graphs for αSMA+ cells or collagen content within the plaque shown in (**B**,**C**), respectively. The plaque stability index (SMC area + collagen area)/(macrophage area + lipid area) and percentage of plaque necrosis are also shown (**D**) and (**E**), respectively. Data are presented as mean ± SEM (*n* = 8 for HFD in (**B**–**E**) and HFD+UA for (**C**–**E**); and *n* = 10 for HFD+UA for (**B**)) with statistical analysis performed using an unpaired Student’s *t*-test (*, *p* ≤ 0.05; **, *p* ≤ 0.01; NS, not significant).

Sections of the aortic root were analysed to determine plaque parameters. UA produced a significant reduction in plaque content (*p* = 0.007), plaque size (*p* = 0.039), and occlusion (*p* = 0.004) without affecting plaque lipid content (Figure 7). UA also produced a significant reduction in the plaque content of macrophages and CD3+ T cells (*p* ≤ 0.001 in both cases) (Figure 8), thereby demonstrating a marked impact in dampening plaque inflammation. In contrast, UA increased plaque α-smooth muscle actin (SMA)+ SMCs and collagen (*p* = 0.005 and *p* = 0.043, respectively) (Figure 9). These changes were associated with a significant increase in the plaque stability index (*p* = 0.002) without any change in plaque necrosis (Figure 9). Overall, these results show that UA attenuates plaque inflammation and produces a stable plaque phenotype. The potential molecular mechanisms underlying such protective changes were investigated further by evaluating other parameters in these animals.

### 3.6. UA Modulates Immune Cell Profile in the Peripheral Blood of LDLr^−/−^ Mice Fed an HFD

There were no significant differences in total weight gain or the weight of either individual white fat depots (i.e., subcutaneous, gonadal, inguinal, and renal) or total white fats between the two groups (Table 1). However, UA supplementation produced a significant increase in the interscapular brown fat (*p* = 0.007) (Table 1). UA also produced significant reductions in the weight of the heart and spleen (*p* = 0.003 and *p* = 0.009, respectively) with no significant changes seen in the weight of the liver and the thymus (Table 1).

UA had no significant effects on the plasma levels of TC, free cholesterol (FC), LDL/VLDL-C, HDL-C, CE, and TG (Table 1). The levels of several SCFA, which are produced by the gut microbiota, in the plasma and the faeces were determined, given their roles in modulating inflammation and atherosclerosis [22]. For the plasma, UA produced a significant increase in the levels of total SCFA (*p* = 0.026), acetic acid (*p* = 0.026), and propionic acid (*p* = 0.024) with no change seen for isobutyric acid (Table 1). Other SCFAs, such as butyric acid, isovaleric acid, and valeric acid, were not detected in the plasma. In the case of the faeces, UA produced a significant increase in total SCFA (*p* = 0.041), acetic acid (*p* = 0.014), and propionic acid (*p* = 0.011), with butyric acid showing a trend towards an increase (*p* = 0.081) (Table 1). No significant changes were seen in isobutyric acid, isovaleric acid, and valeric acid between the two groups (Table 1).

For the peripheral blood lymphoid cells, UA produced a significant reduction in natural killer (NK) cells (*p* = 0.013) without affecting B cells and T cells (CD4+ and CD8+) (Table 1). For myeloid cell populations, UA produced a significant reduction in monocytes (*p* = 0.049), Ly6C^high^ monocytes (*p* = 0.047), Ly6C^low^ monocytes (*p* = 0.015), and granulocytes (*p* = 0.017) (Table 1).

### 3.7. RNA-Seq of the Thoracic Aorta Identifies Key Genes and Pathways That Are Potentially Involved in the Anti-Atherogenic Actions of UA

**Figure 10:**
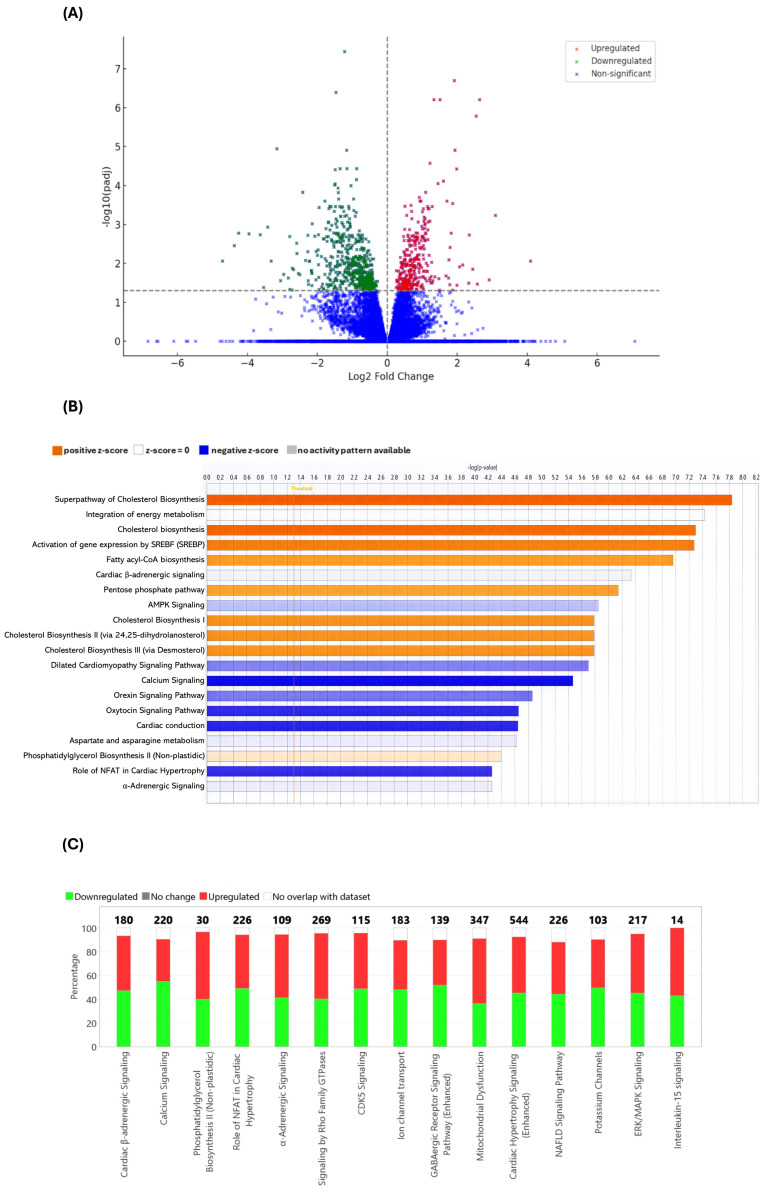
RNA-sequencing of the thoracic aorta identifies key atherosclerosis-associated genes and pathways regulated by UA. (**A**) Volcano plot of DEGs from RNA-seq analysis of the thoracic aorta. Each point represents DEGs, with the *x*-axis showing the log2 fold change in expression levels between the two conditions, and the *y*-axis representing the negative logarithm to the base 10 of the adjusted *p*-value (−log10[adjusted *p*-value]). The blue dots represent non-significant DEGs, red dots represent significantly upregulated DEGs, while green dots represent significantly downregulated DEGs. (**B**) The top 20 most significant canonical pathways regulated by UA intervention. Horizontal bar chart represents pathway enrichment analysis, with the significance of each pathway indicated by the bar’s length. The *x*-axis displays the negative logarithm of the *p*-value (−log[*p*-value]), providing a measure of the significance level for the enrichment of each pathway; longer bars correspond to more significant enrichment. Pathways are listed on the *y*-axis and are colour-coded: orange bars represent pathways with a positive z-score (activation); blue bars indicate pathways with a negative z-score (inhibition); and white bars denote pathways where a z-score cannot be determined. The threshold line denotes the *p*-value cutoff for significance, with bars extending beyond this line considered statistically significant. (**C**) Stacked bar chart detailing the gene expression regulation in atherosclerosis-associated canonical pathways as identified by IPA. Each bar corresponds to a different pathway, listed on the *x*-axis, and is segmented by colour to represent the percentage of genes that are upregulated (red) and downregulated (green) within that pathway. White segments indicate genes that are part of the pathway but not represented in the dataset. Numbers at the top indicate the total number of genes in each pathway (*n* = 4 for each group).

A volcano plot of DEGs from RNA-seq analysis is shown in Figure 10A. There were 867 DEGs that showed a significant difference between the two groups (*p*adj. < 0.05) with 541 downregulated and 326 upregulated. Supplementary Table S1 shows a list of the top 20 upregulated and downregulated genes with their proposed functions. These include genes implicated in the control of metabolism, particularly for carbohydrate and lipids [e.g., Acyl-CoA *thioesterase 11* (*Acot11*), *ATP citrate lyase* (*Acly*), *Thyroid hormone responsive protein* (*Thrsp*), *Solute carrier family 25 member 1* (*Slc25a1*), *ELOVL fatty acid elongase* (*Elovl6*), *Phosphogluconate dehydrogenase* (*Pgd*), *MLX interacting protein-like/Carbohydrate responsive element-binding protein* (*Mlxipl/ChREBP*), *1-Acylglycerol 3-phosphate O-acyltransferase 2* (*Agpat2*), *Glycerol 3-phosphate dehydrogenase 1* (*Gpd1*), *Phospholipase A2 group V* (*Pla2g5*), *Neutral cholesterol ester hydrolase 1* (*Nceh1*), *TBC1 domain family member 1* (*Tbc1d1*)]; signal transduction pathways [e.g., Adenylate *cyclase 10* (*Adcy10*), *A disintegrin and metalloproteinase 19* (*Adam19*), *Ras-related glycolysis inhibitor and calcium channel regulator* (*Rrad*), *Kelch-like family member 32* (*Klhl32*), *Dickkopf Wnt signalling pathway inhibitor 3* (*Dkk3*), *Phosphatidylinositol 4-phosphate 5-kinase type 1 beta* (*Pip5k1b*), *Sorbin and SH3 domain containing 2* (*Sorbs2*)]; and regulation of inflammation [e.g., SH3 *domain binding kinase 1* (*Sbk1*), *Adam19*, *Pla2g5*, ADAMTS-like 2 (*Adamtsl2*), *Dkk3*] (Supplementary Table S1). The 30 most significant terms with the number of genes from GO analysis of DEGs on biological processes, cellular components, and molecular functions are shown in Supplementary Figure S3 and include those implicated in the control of heart physiology, circulation, and fatty acid metabolism. Further analysis of canonical pathways was carried out by IPA to identify the significant pathways influenced by UA (Figure 10B) and those that are potentially implicated in the pathogenesis of atherosclerosis, which may explain, at least in part, the data obtained in this study (Figure 10C and Supplementary Table S2). UA regulated several pathways, including inhibition of potentially pro-atherogenic calcium signalling, the role of nuclear factor of activated T cells (NFAT) in cardiac hypertrophy, α-adrenergic signalling, signalling by Rho family GTPases, cardiac hypertrophy signalling, extracellular signal-regulated kinase (ERK)/mitogen-activated protein kinase (MAPK) signalling, and IL-15 signalling (Figure 10C).

Additional analyses of the RNA-seq data were carried out to further delineate pathway-function relationships. In relation to signalling pathways associated with atherosclerosis, consistent with the anti-inflammatory actions of UA in vitro and in vivo, inhibition of the MCP-1 pathway, a critical axis of inflammation, was predicted. On the other hand, activation was predicted for the peroxisome proliferator-activated receptor α (PPARα)/retinoid X receptor α (RXRα) pathway, suggesting a role in lipid metabolism and energy homeostasis, and the antioxidant action of the Vitamin C pathway, pointing towards an enhancement in cellular antioxidative defences. Further analyses of the regulation of biological functions by IPA also indicated inhibition of cardiovascular disease, particularly myocardial infarction, lipid and carbohydrate metabolism, and nutritional disease (Supplementary Figure S4).

## 4. Discussion

Despite the many beneficial effects associated with pomegranates and their polyphenol PC, the underlying molecular mechanisms remain poorly understood. It is now believed that the protective actions of PC are mediated via urolithins, metabolites produced by the gut microbiota [2]. However, to our knowledge, no studies have so far carried out a comparison of the effects of PC, EA, and different urolithins on key atherosclerosis-associated processes in vitro. This formed the initial focus of this study, where we demonstrate the beneficial effects of PC and its metabolites on several pro-atherogenic processes in monocytes, macrophages, and endothelial cells, though some agent-specific effects were seen (Figure 1, Figure 2, Figure 3, Figure 4, Figure 5 and Figure 6). UA had a pronounced effect, inhibiting several pro-atherogenic processes such as macropinocytosis, ROS production, MCP-1-induced monocytic migration, oxLDL uptake, and pro-inflammatory gene expression (Figure 1, Figure 2, Figure 3, Figure 4, Figure 5 and Figure 6). The actions of UA were therefore also analysed in vivo, where attenuation of plaque burden with a more stable phenotype, improvement of immune cell profile in the peripheral blood, and the production of beneficial SCFA were seen (Figure 7, Figure 8 and Figure 9, Table 1). In addition, RNA-seq of the thoracic aorta identified key genes and pathways for the protective actions of UA that included beneficial effects against oxidative stress, inflammation, and metabolism (Figure 10, Supplementary Tables S1 and S2, and Supplementary Figures S3 and S4). Taken together, these studies provide novel insights into the athero-protective actions of UA together with the potential underlying mechanisms.

The production of ROS is important for the oxidation of LDL [1], and PC, EA, UA, and UC produced a significant reduction in the TBHP-induced cellular ROS production with significant pro-oxidation actions with UB and UD (Figure 1 and Figure 2). The antioxidant and pro-oxidant properties of PC and its metabolites are attributed to the hydroxyl group as well as the lipophilicity of the metabolite, but also depend on the assay system and conditions [23,24]. The pathways responsible for the modulation of ROS production by PC and its metabolites are not fully understood. However, PC protected against doxorubicin-induced cardiotoxicity by suppressing ROS generation and activating the nuclear factor erythroid 2-related factor (Nrf2) signalling pathway, which protects against oxidative stress and induces the expression of antioxidant and phase II detoxification enzymes [25]. The chemokine MCP-1 plays a critical role in the recruitment of monocytes that then differentiate into macrophages and transform into foam cells [1]. PC and its metabolites (EA, UA, and UC) decreased the MCP-1-driven monocytic migration (Figure 5A). PC has been previously demonstrated to decrease the MCP-1-induced monocytic migration [26]. The macrophage uptake of LDL/modified LDL is regulated by several processes, including macropinocytosis and receptor-mediated endocytosis [1]. To our knowledge, this is the first study that has investigated the effects of PC and its metabolites on macrophage macropinocytosis activity, where an inhibition was seen (Figure 3). Indeed, inhibition of macropinocytosis reduced atherosclerotic lesion development in mice lacking LDLr^−/−^ and ApoE^−/−^ [27]. Finally, the uptake of Dil-oxLDL, which is predominantly mediated via receptor-mediated endocytosis, was markedly inhibited by PC and its metabolites (Figure 5B). Previous studies have shown that PC, EA, UA, and UB attenuate cholesterol uptake by macrophages [3,7,28].

IL-1β is a key pro-atherogenic cytokine that is secreted following the activation of the inflammasome pathway [1,14]. PC and UA significantly inhibited IL-1β secretion in the THP-1 model system, whereas EA and UC significantly increased this (Figure 6A). PC has been shown previously to inhibit the release of IL-1β in macrophages [29]. In addition to the secretion of IL-1β, PC, UA, and UC decrease the expression of MCP-1 and ICAM-1 induced by the pro-atherogenic cytokine IFN-γ (Figure 6B,C). Again, PC has been shown to inhibit the IFN-γ-induced expression of MCP-1 and ICAM-1 in THP-1 macrophages [26]. This finding may be linked to the antioxidant property of PC and its metabolites (UA and UC) (Figure 1 and Figure 2). Indeed, ROS production has been associated with increased production of pro-inflammatory cytokines and factors, including activation of inflammasome pathways [30]. TNF-α is a critical regulator of endothelial cell dysfunction [1]. PC and its metabolites inhibited the expression of MCP-1 and ICAM-1 in HAECs treated with this cytokine (Figure 6D,E). This finding correlates with a previous study that showed PC inhibition of TNF-α-induced MCP-1 and ICAM-1 expression in human umbilical vein endothelial cells (HUVECs) [31]. Overall, these results underscore the potent anti-inflammatory action of PC and some of its metabolites.

Analysis of fat depots showed that UA supplementation significantly increased the content of the brown adipose tissue (BAT) (Table 1). This is consistent with previously noted beneficial metabolic effects of UA, together with stimulation of mitochondrial biogenesis in brown adipocytes in vitro [32]. Increased BAT activity has many protective actions. For example, BAT actively consumes glucose and lipids to produce heat, thereby reducing the levels of circulating lipids and glucose [33]. In addition, the thermogenic activity of BAT is associated with anti-inflammatory effects [34].

UA supplementation had no significant effects on plasma levels of TC, VLDL/LDL-C, TG, or HDL-C (Table 1), which may correlate with no changes seen in plaque lipid content (Figure 7E). Indeed, UA produced no significant changes in plasma TC and TG levels in HFD-fed DBA2J mice [35] or in HDL-C levels in HFD-fed rats [36]. There are many other published studies that also show plaque attenuation that is independent of any changes in plasma lipid/lipoprotein profiles or even when the plasma cholesterol levels are increased [37,38,39,40]. For example, deficiency of *interleukin-18* or *hepatic lipase* attenuates atherosclerosis despite an increase in plasma cholesterol levels [39,40]. Multiple mechanisms have been proposed in such studies, including antioxidant actions, suppression of vascular inflammation, and increased plaque stability via modulation of SMC biology [37,38,39,40]. Interestingly, many such changes were produced by UA in the current study. Thus, UA attenuated the TBHP-induced ROS production in human monocytes and macrophages (Figure 1, Figure 2 and Figure 4), inhibited pro-inflammatory gene expression in human macrophages and endothelial cells (Figure 6), and increased markers of plaque stability, including levels of SMCs (Figure 9).

SCFAs are primarily produced by the microbial fermentation of dietary fibres in the gut and play crucial roles in maintaining gut health, modulating immune responses, and providing energy to the host [41]. UA is also a metabolite derived from PC and EA, and its production is dependent on gut microbial metabolism; similarly, UA can influence the composition and function of the gut microbiota [2]. The concentrations of total SCFAs, acetic acid, and propionic acid in both the plasma and the faeces were increased by UA supplementation with butyric acid, showing a trend towards an increase in the faeces (Table 1). Overall, these results suggest that UA supplementation potentially modulates the gut microbiota to promote the fermentation of dietary fibres, thereby leading to increased SCFAs production. However, this needs to be confirmed via an in-depth analysis of the gut microbiota. The increased production of SCFAs has potential health benefits, as they have been shown to exert beneficial effects on metabolic health, including improved glucose homeostasis and insulin sensitivity, together with reduced atherosclerotic lesion development [22,42].

Analysis of the peripheral blood showed that UA supplementation produced a significant attenuation in the levels of NK cells together with monocytes (both Ly6C^high^ and Ly6C^low^) and granulocytes (Table 1). These findings are consistent with the anti-inflammatory properties of UA, as NK cells, monocytes, particularly the Ly6C^high^ subtype, and granulocytes, specifically neutrophils, all promote atherosclerosis by producing pro-inflammatory factors [43]. Reduction in these cells has also been associated with improved plaque stability [44,45]. The anti-inflammatory actions of UA were also seen at the level of aortic root atherosclerotic plaques, where there were reductions in plaque size together with the content of macrophages and CD3+ T cells (Figure 7 and Figure 8). Macrophages and CD3+ T cells produce a range of pro-inflammatory factors that cause plaque destabilisation via increased apoptosis of SMCs and degradation of fibrous cap proteins [43,44,45,46]. Indeed, UA caused plaque stabilisation by increasing the content of SMCs and collagen (Figure 9). One such pro-inflammatory factor is the cytokine IFN-γ, whose actions were attenuated by UA in vitro (Figure 6B,C). Indeed, IFN-γ plays a critical role in the differentiation of monocytes into macrophages and promotes apoptosis of SMCs and expression/activities of matrix metalloproteinases [1,47]. Thus, previous studies on ApoE^−/−^ mice showed that deficiency of IFN-γ led to decreased levels of macrophages and increased collagen content in the plaques [1,47]. Conversely, the opposite effects were seen following administration of exogenous IFN-γ, which produced adverse effects [1,47].

The low bioavailability of PC and EA is well established, and only a limited number of studies have examined this in relation to urolithins [2,48,49,50]. Nevertheless, the available evidence indicates that the bioavailability of urolithins is highly variable and depends strongly on the composition of the gut microbiota, the specific metabolite, its concentration, and the dietary source of ET [2,48,49,50]. Following absorption, urolithins undergo extensive hepatic metabolism to form glucuronide and sulphate conjugates, which predominate in the circulation and may exhibit biological activities distinct from those of the free compounds [48,49,50]. Typically, total plasma concentrations of urolithins range from approximately 0.024 to 35 μM, with urinary levels reaching up to 50 μM [48,49,50]. These factors complicate efforts to directly interpret the physiological relevance of in vitro concentrations or in vivo doses used in experimental studies. It should also be noted that in vitro studies often utilise a single cell type, and observed effects may not fully translate to the more complex in vivo environment where multiple cell types and mediators interact. Despite these potential limitations, the concentrations used in our study are consistent with those employed in the published literature. For example, an early study demonstrating that UA prolongs the lifespan in *C. elegans* and enhances muscle function in rodents used concentrations of up to 50 μM in worms and C2C12 myoblasts, and 50 mg/kg/day in mice [17]. Significant effects were also reported at 50 μM in a previous in vitro study on atherosclerosis [5]. The 50 mg/kg/day dose in mice has been widely employed in subsequent studies [17,18,19,20], including those examining improvements in muscle function that were first observed in humans [17,19]. Human clinical trials commonly administer 1000 mg/day for periods ranging from 28 days to four months [51,52].

RNA-seq of the thoracic aorta revealed several canonical pathways that play important roles in the control of inflammation and key processes associated with ASCVD (Figure 10, Supplementary Figures S3 and S4, Supplementary Tables S1 and S2). Future studies should seek to confirm the regulation of these pathways by biochemical assays and their roles by genetic (e.g., knockdown, knockout, or overexpression) or pharmacological (e.g., inhibitors or agonists) approaches. The cardiac β-adrenergic signalling pathway was predicted to be inhibited by UA (Supplementary Table S2), and attenuation of this pathway by β-blockers confers multiple cardiovascular benefits: reduced cardiac workload; lowered blood pressure; dampened inflammation and oxidative stress; and improved endothelial function [53]. Calcification is associated with advanced, unstable plaques [54] and, consistent with its plaque-stabilising actions, UA was predicted to inhibit calcium signalling pathways. In addition, calcium signalling pathways have been implicated in SMC proliferation and phenotypic switch to a calcification-prone phenotype, endothelial dysfunction, and inflammatory processes within atherosclerotic plaques [55]. In addition, imbalances in calcium homeostasis can adversely affect mitochondrial dynamics, thereby promoting oxidative stress and inflammation [56]. The NFAT pathway, which has been implicated in the progression of cardiac hypertrophy, together with foam cell formation, inflammation, and vascular calcification [57,58], was also predicted to be inhibited by UA.

Several other key pathways driving inflammation and atherosclerosis were also predicted to be regulated by UA. Thus, the MCP-1 pathway, which orchestrates the recruitment and infiltration of monocytes to the endothelial layer [1,47], was inhibited and is consistent with the UA-mediated attenuation of chemokine-driven monocytic migration in vitro (Figure 5A). On the other hand, the PPAR/RXR pathway was activated by UA. This pathway plays a pivotal role in the regulation of lipid metabolism, including increased fatty acid β-oxidation and improved HDL function, together with inhibition of inflammation [59,60]. Indeed, pomegranate flower extracts improved cardiac lipid metabolism in diabetic rats by activating PPAR, lowering circulating lipids, and inhibiting cardiac lipid uptake [61]. PPARα also plays a role in glucose metabolism, so its activation may help in maintaining glucose homeostasis, which is often disrupted in metabolic disorders like diabetes and obesity, commonly associated with atherosclerosis [62]. UA also activates the antioxidant action of Vitamin C on cellular signalling, which is consistent with its potent antioxidant activities (Figure 1, Figure 2 and Figure 4), and also attenuates inflammation [63,64]. For example, vitamin C inhibits several inflammation-associated pathways [63,64].

The effect of UA on atherosclerosis has, in part, been investigated recently in HUVECs in vitro and male ApoE^−/−^ mice in vivo, though RNA-seq was only performed on the former [5]. Although there were several similar outcomes to the current study, there were many differences as well. Thus, the anti-inflammatory actions of UA were seen in HUVECs, as we have observed here with HAECs (Figure 6). However, in sections of the aortic root, whilst reduced macrophage content was observed, no changes were seen in relation to plaque content together with those of SMC and collagen, though these changed when the brachiocephalic artery was analysed [5]. The precise reasons for the differences are not clear, though it must be noted that the ApoE^−/−^ mouse is a more aggressive model compared to LDLr^−/−,^ with disease formation seen following feeding of a chow diet, which is sped up with an HFD, and increases seen in plasma VLDL and chylomicron fractions compared to LDL-C in humans [9]. In addition, ApoE is involved in the control of several key processes, including inflammation and SMC proliferation, which may therefore impact plaque development in ApoE^−/−^ mice [9]. In contrast, LDLr^−/−^ mice develop atherosclerosis when fed an HFD, akin to diet-induced atherosclerosis in humans, and have increases in plasma LDL-C similar to individuals with familial hypercholesterolemia [9].

There are several limitations associated with the present study. First, although sex-specific differences in ASCVD have been reported [65], as with the previous published study in ApoE^−/−^ mice [5], the current investigation was conducted exclusively in male mice. Second, only a single dose of UA and a single intervention time point were evaluated. A dose–response analysis and assessment of UA effects on more advanced plaques (e.g., after 24 weeks of HFD feeding) would have strengthened the findings. Third, the pharmacokinetic properties of UA—such as absorption, distribution, metabolism, and excretion—were not examined. Fourth, gut microbiome profiling should be undertaken to contextualise the UA-induced changes observed in plasma and faecal SCFA levels (Table 1). Fifth, the alterations in gene expression and pathway modulation following UA treatment require further validation using additional assays, as well as mechanistic investigation through gene/pathway inhibition or overexpression. Finally, although dose–response experiments were performed in in vitro studies, the concentrations employed were generally higher than those typically observed under physiological conditions [48,49,50].

## 5. Conclusions

This study provides new insights into the protective actions of PC and its metabolites on atherosclerosis, together with the underlying molecular mechanisms. Many pro-atherogenic processes, including ROS production, pro-inflammatory gene expression, chemokine-driven monocytic migration, and activation of the inflammasome, were inhibited in vitro, though some agent-specific responses were also seen. UA had the most pronounced effects, attenuated plaque content and inflammation in vivo, and produced a stable plaque phenotype. The anti-inflammatory actions of UA in vivo extended to immune cells in the peripheral blood. Future studies should investigate whether the anti-inflammatory and plaque-stabilising actions of UA extend to animal models of regression of existing/established atherosclerotic plaques and in clinical trials together with the roles of key genes and pathways identified using knockdown, knockout, or overexpression approaches.

## Figures and Tables

**Table 1:** The effects of UA on atherosclerosis-associated risk factors in LDLr^−/−^ fed HFD.

		HFD		HFD + UA	*p*-Value
	*N*	Mean ± SEM	*N*	Mean ± SEM	
Overall weight gain [g]	19	6.35 ± 0.69	19	7.34 ± 0.85	NS
Adipose tissue deposits [g]					
Total	15	0.048 ± 0.003	15	0.049 ± 0.004	NS
Total white	15	0.045 ± 0.005	15	0.044 ± 0.003	NS
Brown	15	0.003 ± 0.0002	15	0.004 ± 0.0002	0.007
Subcutaneous	15	0.021 ± 0.002	15	0.020 ± 0.001	NS
Gonadal	15	0.018 ± 0.002	15	0.019 ± 0.001	NS
Inguinal	15	0.002 ± 0.0001	15	0.001 ± 0.0001	NS
Renal	15	0.003 ± 0.0004	15	0.003 ± 0.0003	NS
Organ weights [g]					
Heart	19	0.005 ± 0.0002	19	0.004 ± 0.0001	0.003
Liver	19	0.047 ± 0.0008	19	0.048 ± 0.001	NS
Spleen	19	0.004 ± 0.0003	18	0.003 ± 0.0002	0.009
Thymus	19	0.001 ± 0.0001	18	0.001 ± 0.0001	NS
Plasma lipids [mg/dL]					
TG	15	71.5 ± 5.39	15	71.4 ± 4.36	NS
TC	15	637.3 ± 43.1	15	529.5 ± 34.5	NS
FC	15	440.0 ± 26.4	15	404.2 ± 37.7	NS
HDL-C	15	67.8 ± 1.56	15	72.9 ± 3.28	NS
LDL/VLDL-C	13	328.5 ± 9.2	13	376.6 ± 9.9	NS
CE	13	161.0 ± 16.3	14	152.8 ± 18.3	NS
Short-chain fatty acids (SCFA), plasma (μg/mL)					
Total SCFA	8	0.9 ± 0.04	8	1.1 ± 0.04	0.026
Acetic acid	9	0.6 ± 0.03	9	0.7 ± 0.04	0.026
Propionic acid	9	0.17 ± 0.01	9	0.2 ± 0.01	0.024
Isobutyric acid	10	0.1 ± 0.004	9	0.1 ± 0.006	NS
SCFA, faeces (μg/g)					
Total SCFA	9	37.3 ± 3.6	9	52.5 ± 5.3	0.041
Acetic acid	9	20.6 ± 2.02	9	30.3 ± 2.61	0.014
Propionic acid	9	6.2 ± 0.67	9	8.9 ± 0.55	0.011
Isobutyric acid	10	0.8 ± 0.11	10	0.8 ± 0.15	NS
Butyric acid	8	7.9 ± 0.94	8	13.2 ± 2.49	0.081
Isovaleric acid	10	0.9 ± 0.14	10	1.1 ± 0.21	NS
Valeric acid	8	0.9 ± 0.09	8	1.5 ± 0.42	NS
Peripheral blood—Lymphoid cells (frequency of nucleated cells)					
B cells	12	41.8 ± 3.14	12	36.8 ± 2.64	NS
T cells	12	16.1 ± 0.88	12	16.2 ± 0.63	NS
CD4	11	7.0 ± 0.82	11	7.2 ± 0.22	NS
CD8	12	7.7 ± 0.29	12	9.1 ± 0.36	NS
NK cells	12	1.5 ± 0.21	12	0.9 ± 0.07	0.013
Peripheral blood—Myeloid cells (frequency of nucleated cells)					
Monocytes (CD115^+^)	10	4.8 ± 0.89	12	3.0 ± 0.13	0.049
Ly6C^high^ monocytes	10	2.9 ± 0.86	11	1.1 ± 0.13	0.047
Ly6C^low^ monocytes	10	1.3 ± 0.08	10	1.0 ± 0.06	0.015
Granulocytes	10	19.3 ± 4.81	13	7.6 ± 2.57	0.017

CE, cholesteryl esters; FC, free cholesterol; HDL-C, high-density lipoprotein cholesterol; LDL/VLDL-C, low-density lipoprotein/very low-density lipoprotein cholesterol; NK, natural killer; NS, not significant; TC, total cholesterol; TG, triacylglycerol.

## Data Availability

RNA-sequencing data have been submitted to the GEO repository (GSE311266). The other original contributions present in this study are included in the article and Supplementary Materials. Further inquiries can be directed to the corresponding author.

## Supplementary Materials

The following supporting information can be downloaded at: https://www.mdpi.com/article/10.3390/antiox15040507/s1.

## Abbreviations

The following abbreviations are used in this manuscript:

ASCVD	Atherosclerotic cardiovascular disease
Adam19	A disintegrin and metalloproteinase 19
Adcy10	Adenylate cyclase 10
Acly	ATP citrate lyase
Acot11	Acyl-CoA thioesterase 11
Adamstsl2	ADAMTS-like 2
Agpat2	1-Acylglycerol 3-phosphate O-acyltransferase 2
ANOVA	One-way analysis of variance
ApoE	Apolipoprotein E
BAT	Brown adipose tissue
CE	Cholesteryl esters
DCFDA	2′, 7′-dichlorofluorescin diacetate
DEGs	Differentially expressed genes
DMSO	Dimethyl sulfoxide
Dkk3	Dickkopf Wnt signalling pathway inhibitor 3
EA	Ellagic acid
ECM	Extracellular matrix
Elovl6	ELOVL fatty acid elongase
ERK	Extracellular signal-regulated kinase
ET	Ellagitannins
FC	Free cholesterol
GAPDH	Glyceraldehyde 3-phosphate dehydrogenase
GC	Gas chromatography
GO	Gene ontology
Gpd1	Glycerol 3-phosphate dehydrogenase 1
HAEC	Human aortic endothelial cells
HDL	High-density lipoprotein
HDL-C	High-density lipoprotein cholesterol
HFD	High-fat diet
HMDM	Human monocyte-derived macrophages
HUVECs	Human umbilical vein endothelial cells
ICAM-1	Intercellular adhesion molecule-1
IFN-γ	Interferon-γ
IL	Interleukin
IPA	Ingenuity Pathway Analysis
KEGG	Kyoto Encyclopedia of Genes and Genomes
Klhl32	Kelch-like family member 32
LDH	Lactate dehydrogenase
LDL	Low-density lipoprotein
LDL/VLDL-C	Low-density lipoprotein/very low-density lipoprotein cholesterol
LDLr^−/−^	Low-density lipoprotein receptor-deficient mice
LY	Lucifer Yellow
MAPK	Mitogen-activated protein kinase
MCP-1	Monocyte chemotactic protein-1
Mlxipl/ChREBP	MLX interacting protein-like/Carbohydrate responsive element binding protein
Nceh1	Neutral cholesterol ester hydrolase 1
NFAT	Nuclear factor of activated T cells
NK	Natural killer
Nrf2	Nuclear factor erythroid-2 related factor
NS	Not significant
OCT	Optimum cutting temperature
ORO	Oil red O
oxLDL	Oxidised LDL
Padj	Adjusted *p*-value
PBS	Phosphate-buffered saline
PC	Punicalagin
Pgd	Phosphogluconate dehydrogenase
Pip5k1b	Phosphatidyl-inositol 4-phosphate 5-kinase type 1 beta
Pla2g5	Phospholipase A2 group V
PMA	Phorbol 12-myristate 13-acetate
PPAR	Peroxisome proliferators-activated receptors
RNA-seq	RNA-sequencing
ROS	Reactive oxygen species
Rrad	Ras-related glycolysis inhibitor and calcium channel regulator
RT-qPCR	Real-time quantitative polymerase chain reaction
RXR	Retinoid X receptor
Sbk1	SH3 domain binding kinase 1
SCFA	Short-chain fatty acids
Scl25a1	Solute carrier family 25 member 1
SEM	Standard error of the mean
SMA	Smooth muscle actin
SMC	Smooth muscle cells
Sorbs2	Sorbin and SH3 domain-containing 2
Tbc1d1	TBC1 domain family member 1
TBHP	Tert-butyl hydroperoxide
TC	Total cholesterol
TG	Triacylglycerol
Thrsp	Thyroid hormone-responsive protein
TNF-α	Tumour necrosis factor-α
U	Urolithin
UA	Urolithin A
VLDL	Very low-density lipoprotein
