# Supplementary material for: Anti-Atherogenic Actions of Pomegranate Polyphenol Punicalagin and Its Metabolites: In Vitro Effects on Vascular Cells and In Vivo Atheroprotection by Urolithin A via Anti-Inflammatory and Plaque-Stabilising Mechanisms
Source: Antioxidants (Basel). 2026 Apr 20;15(4):507. doi: 10.3390/antiox15040507 (PMC13113821; doi:10.3390/antiox15040507)
Supplement: Supplementary file 1 [file antioxidants-15-00507-s001.zip › antioxidants-4213104-supplementary.pdf]

Anti-atherogenic Actions of Pomegranate Polyphenol Punicalagin and Its Metabolites: In Vitro Effects on Vascular Cells and In Vivo Atheroprotection by Urolithin A via Anti-Inflammatory and Plaque-Stabilising Mechanisms

by

Sulaiman Alalawi[1,2], Daniah Rifqi[1], Alaa Alhamadi[1], Reem Alotibi[1], Fahad Alradi[1], Nouf Alshehri[1], Yee-Hung Chan[1], Jing Chen[1], Faizah Albalawi[1], Sarab Taha[3], Nabras Al-Mahrami[4], Irina A. Guschina[1], Timothy R. Hughes[5] and Dipak P. Ramji[1*]

[1]Cardiff School of Biosciences, Cardiff University, Sir Martin Evans Building, Museum Avenue, Cardiff CF10 3AX, UK.

[2] Ministry of Health, P.O. Box 393, Muscat 100, Oman.

[3]European Cancer Stem Cell Research Institute, Cardiff School of Biosciences, Cardiff University, Hadyn Ellis Building, Maindy Road, Cardiff CF24 4HQ, UK.

[4]Medical Laboratory Sciences, Health Sciences, Oman College of Health Sciences, P. O. Box 3720, Muscat 112, Oman

[5]Division of Infection and Immunity, Henry Wellcome Building, School of Medicine, Cardiff University, Heath Park, Cardiff CF14 4XN, UK.

***Corresponding author:** Professor Dipak P. Ramji, Cardiff School of Biosciences, Cardiff University, Sir Martin Evans Building, Museum Avenue, Cardiff CF10 3AX, UK. Tel: 0044 (0)29 20876753; Fax: 0044 (0)29 20874116; Email: Ramji@Cardiff.ac.uk

Supplementary Table S1. List of top 20 significant upregulated and downregulated DEGs

Gene ID	Gene name	Log2Fold Change	Padj	Function of protein
**Upregulated**				
ENSMUSG00000006784	*Ttc25*	1.91139	2.01E-07	Protein-protein interactions and signal transduction
ENSMUSG00000034853	*Acot11*	1.33277	6.17E-07	Catalyses the conversion of activated fatty acids to the corresponding non-esterified fatty acids and coenzyme A
ENSMUSG00000020917	*Acly*	1.50498	6.17E-07	Catalysis the synthesis of acetyl-CoA from citrate
ENSMUSG00000031449	*Atp4b*	2.63393	6.18E-07	The beta subunit of gastric H^{+}/K^{+} ATPase in apical membrane of parietal cells
ENSMUSG00000028976	*Slc2a5*	2.53732	1.63E-06	Fructose uptake by the small intestine
ENSMUSG00000026567	*Adcy10*	1.93069	1.23E-05	Soluble form of adenyate cyclase involved in cell signalling
ENSMUSG00000035686	*Thrsp*	1.2162	2.64E-05	Regulation of lipogenesis
ENSMUSG00000021509	*Slc25a48*	1.60038	7.68E-05	Mitochondrial inner-membrane carrier protein that regulates choline import and metabolism
ENSMUSG00000046167	*Gldn*	1.44446	8.85e-05	Efficient transmission of nerve impulses
ENSMUSG00000041220	*Elovl6*	1.09481	0.000149	Elongation of long-chain fatty acids
ENSMUSG00000038009	*Dnajc22*	1.70093	0.000248	Co-chaperone
ENSMUSG00000031936	*Hephl1*	1.86425	0.000286	Regulation of intracellular iron homeostasis
ENSMUSG00000003528	*Slc25a1*	1.28504	0.000346	Regulates citrate transport across inner membrane of mitochondria
ENSMUSG00000028961	*Pgd*	1.15498	0.000346	Catalyses the oxidative decarboxylation of 6-

				phosphogluconate to ribulose 5-phosphate in the pentose phosphate pathway
ENSMUSG00000042978	*Sbk1*	1.02643	0.000389	Cell signalling
ENSMUSG00000005373	*Mlxipl*	1.19379	0.000389	Carbohydrate-responsive element binding protein that regulates metabolism, particularly glycolysis and lipogenesis
ENSMUSG00000001420	*Tmem79*	1.16059	0.00039	Encodes a transmembrane protein linked to atopic dermatitis and skin barrier function
ENSMUSG00000026922	*Agpat2*	1.00746	0.000466	Encodes an enzyme that is involved in the glycerolipid biosynthesis pathway
ENSMUSG00000023019	*Gpd1*	1.05015	0.000648	Encodes an enzyme that is pivotal in carbohydrate and lipid metabolism
ENSMUSG00000059040	*Eno1b*	1.0754	0.000699	The precise function is not known
**Downregulated**				
ENSMUSG00000011256	*Adam19*	-1.22294	3.58E-08	Encodes metalloprotease implicated in cell-cell and cell-matrix interactions, signalling and development
ENSMUSG00000031880	*Rrad*	-1.46738	4.02E-07	Regulates intracellular signalling, metabolism and function of the cardiovascular system
ENSMUSG00000105168	*Gm30735*	-3.16306	1.12E-05	The precise function is not known
ENSMUSG00000022270	*Retreg1*	-1.1607	1.23E-05	Regulates endoplasmic reticulum (ER)-autophagy, maintains ER

				homeostasis and affects neuropathy
ENSMUSG00000040387	*Klhl32*	-1.35422	3.66E-05	Implicated in protein ubiquitination and regulation of cell signalling
ENSMUSG00000048967	*Yjefn3*	-1.15673	3.66E-05	The precise function is not known
ENSMUSG00000041193	*Pla2g5*	-1.48973	3.88E-05	Member of the secretory phospholipase A2 family. The encoded enzyme catalyses the hydrolysis of membrane phospholipids to generate lysophospholipids and free fatty acids
ENSMUSG00000036040	*Adamtsl2*	-1.48999	9.05E-05	Encoded protein functions as a secreted glycoprotein that binds the cell surface and the extracellular matrix
ENSMUSG00000027698	*Nceh1*	-1.51071	9.66E-05	Encodes an enzyme essential for hydrolysis of cholesteryl esters and regulation of cellular lipid homeostasis
ENSMUSG00000040260	*Daam2*	-1.04468	0.00010	The encoded protein plays an important role in the Wnt signalling pathway
ENSMUSG00000030772	*Dkk3*	-1.40759	0.00011	Modulates Wnt signalling, affects differentiation and may suppress tumours
ENSMUSG00000107653	*Gm31520*	-2.41798	0.00014	The precise function is not known
ENSMUSG00000024867	*Pip5k1b*	-1.4045	0.00015	Regulator of cytoskeletal dynamics and signal transduction
ENSMUSG00000022037	*Clu*	-1.39578	0.00022	Encodes clusterin, involved in chaperoning,

				apoptosis and lipid transport
ENSMUSG00000044006	*Cilp2*	-1.78965	0.00024	Mediates the interaction between specific membranous organelles and microtubules
ENSMUSG00000029174	*Tbc1d1*	-1.64944	0.00033	Regulates glucose uptake, energy homeostasis and metabolism in muscles
ENSMUSG00000026208	*Des*	-1.10385	0.00033	Encodes desmin, essential for muscle structure and function
ENSMUSG00000053469	*Tg*	-1.48428	0.00034	Encodes thyroglobulin, essential for thyroid hormone synthesis and storage
ENSMUSG00000031626	*Sorbs2*	-1.36298	0.00034	Involved in cytoskeletal, cardiac structure and insulin signalling
ENSMUSG00000078670	*Fam174b*	-1.28099	0.00034	The precise function is not known

Abbreviations: *Acly*, ATP citrate lyase; *Acot11*, Acyl-CoA thioesterase 11; *Adam19*, A disintegrin and metalloproteinase domain 19; *Adamtsl2*, ADAMTS like 2; *Adcy10*, Adenylate cyclase 10; *Agpat2*, 1-Acylglycerol-3-phosphate O-acyltransferase 2; *Atp4b*, ATPase H^+/K^+ transporting subunit beta; *Cilp2*, Cartilage intermediate layer protein 2; *Clu*, Clusterin; *Daam2*, Dishevelled associated activator of morphogenesis 2; *Des*, Desmin; *Dkk3*, Dickkopf Wnt signalling pathway inhibitor 3; *Dnajc22*, DnaJ heat shock protein family (Hsp40) member C22; *Elovl6*, ELOVL fatty acid elongase 6; *Eno1b*, Enolase 1B (beta-enolase); *Fam174b*, Family with sequence similarity 174 member B; *Gldn*, Gliomedin; *Gm31520*, Predicted gene 31520; *Hephl1*, Hephaestin like 1; *Gm30735*, Predicted gene 30735; *Gpd1*, Glycerol-3-phosphate dehydrogenase 1; *Klhl32*, Kelch like family member 32; *Mlxipl*, MLX interacting protein like (ChREBP); *Nceh1*, Neutral cholesterol ester hydrolase 1; *Pip5k1b*, Phosphatidylinositol-4-phosphate 5-kinase type 1 beta; *Pla2g5*, Phospholipase A2 group V; *Pgd*, Phosphogluconate dehydrogenase; *Retreg1*, Reticulophagy regulator 1; *Rrad*, Ras-related glycolysis inhibitor and calcium channel regulator; *Sbk1*, SH3 Domain binding kinase 1; *Slc25a1*, Solute carrier family 25 member 1 (CIC); *Slc2a5*, Solute carrier family 2 member 5 (GLUT5); *Slc25a48*, Solute carrier family 25 member 48; *Sorbs2*, Sorbin and SH3 domain containing 2; *Tbc1d1*, TBC1 domain family member 1; *Tg*, Thyroglobulin; *Thrsp*, Thyroid hormone responsive (Spot14); *Tmem79*, Transmembrane protein 79; *Ttc25*, Tetratricopeptide repeat domain 25; *Yjefn3*, YjeF N-terminal domain containing 3.

Supplementary Table S2 UA regulated canonical pathways and associated genes

Pathways	p-value	z-score	Associated genes
Cardiac β-adrenergic signalling	-4.65-E07	-0.277	*Adcy10, Akap6, Cacna1c, Cacna1d, Cacna2d3, Cacnb2, Ednra, Itpr1, Itpr2, Itpr3, Ryr2*
Calcium signalling	3.42E-06	-3.606	*Asph, Cacna1c, Cacna1d, Cacna2d3, Cacnb2, Camk1d, Camk2a, Camk2d, Cav1, Cav2, Cav3, Ednra, Itpr1, Itpr2, Itpr3, Plcb1, Pln, Prkca, Prkcb, Ryr2, Slc8a1, Trdn*
Phosphatidylglycerol biosynthesis II (non-plastidic)	4.02E-05	1.000	*Agpat2, Cds1, Cds2, Gpd1, Gpd2, Pnpla3, Ptpmt1*
Role of NFAT in cardiac hypertrophy	5.53E-05	-2.668	*Adcy10, Cacna1c, Cacna1d, Cacna2d3, Cacnb2, Camk1d, Camk2a, Camk2d, Cav1, Cav2, Cav3, Ednra, Itpr1, Itpr2, Itpr3, Nfatc1, Nfatc2, Nfatc3, Nfatc4, Plcb1, Pln, Prkca, Prkcb, Ryr2, Slc8a1, Trdn*
α-Adrenergic signalling	5.56E-05	-0.447	*Adcy10, Gna13, Gng12, Gng2, Gng4, Gys2, Phkg1, Prkag2, Prkar1a, Prkar2b, Prkca, Prkcb*
Signalling by Rho family GTPases	7.84E-0.5	-1.069	*Cdc42ep4, Cdh2, Cdh20, Cfl2, Des, Gna13, Gng12, Gng2, Gng4, Ppp1ca, Prkca, Prkcb, Rhoa, Rhob, Rhoj, Rhou, Rock1, Rock2*
CDK5 signalling	9.72E-05	0.577	*Adcy10, Cables1, Lama2, Mapk12, Mapk4, Ntrk2, Ppm1j, Ppp1ca, Prkca, Prkcb*
Ion channel transport	1.08E-04	-2.183	*Ano4, Asph, Atp11a, Atp1a1, Atp1b1, Atp1b3, Atp4b, Atp6v1b2, Cacna1c, Cacna1d, Cacna2d3, Cacnb2, Scn1b, Scn2b, Scn3b, Scn4b, Slc8a1, Tpcn2*
GABAergic receptor signalling pathway (enhanced)	1.85E-04	0.535	*Abat, Adcy10, Cacna1c, Cacna1d, Cacna2d3, Cacnb2, Gna13, Gng12, Gng2,*

			Gng4, Ppp1ca, Prkca, Prkcb
Mitochondrial dysfunction	2E-04	1.000	*Adcy10, Atp1a1, Atp1b1, Atp1b3, Atpaf1, Atpaf2, Cacna1c, Cacna1d, Cacna2d3, Cacnb2, Ednra, Itpr1, Itpr2, Itpr3, Nd1, Nd2, Nd3, Nd4, Nd4l, Nd5, Nd6, Ryr2, Slc25a4, Slc25a5, Slc25a6, Slc8a1, Trdn*
Cardiac hypertrophy signalling (Enhanced)	2.48E-04	-2.887	*Adcy10, Adra1b, Agt, Atf6, Cacna1c, Cacna1d, Cacna2d3, Cacnb2, Camk1d, Camk2a, Camk2d, Cav1, Cav2, Cav3, Ednra, Itpr1, Itpr2, Itpr3, Nfatc1, Nfatc2, Nfatc3, Nfatc4, Plcb1, Pln, Prkca, Prkcb, Ryr2, Slc8a1, Trdn*
NAFLD signalling pathway	4.67E-04	0.471	*Acaca, Atf6, Fasn, Gys2, Il15, Mapk12, Mlxipl, Pnpla3, Prkag2, Prkar1a, Prkar2b, Srebf1*
Potassium channels	5.37E-04	-0.905	*Gng12, Gng2, Gng4, Kcna4, Kcnab1, Kcnb1, Kcng4, Kcnh2, Kcnj3, Kcnk3, Kcnq1, Scn1b, Scn2b, Scn3b, Scn4b*
ERK/MAPK signalling	7.99E-04	-1.604	*Creb3l2, Grb2, Hspb2, Itga8, Itgb6, Pak6, Pla2g5, Ppm1j, Ppp1ca, Prkca, Prkcb*
Interleukin-15 signalling	8.63E-04	-2.000	*Grb2, Il15, Stat3, Stat5b*

Abbreviations: *Abat, 4-aminobutyrate aminotransferase; Acaca, Acetyl-CoA carboxylase alpha; Adcy10, Adenylate cyclase 10; Adra1b, Adrenoceptor alpha 1B; Agpat2, 1-acylglycerol-3-phosphate O-acyltransferase 2; Agt, Angiotensinogen; Akap6, A-kinase anchoring protein 6; Ano4, Anoctamin 4; Asph, Aspartate beta-hydroxylase; Atf6, Activating transcription factor 6; Atp11a, ATPase phospholipid transporting 11A; Atp1a1, ATPase* Na^+/K^+ *transporting subunit alpha 1; Atp1b1, ATPase* Na^+/K^+ *transporting subunit beta 1; Atp1b3, ATPase* Na^+/K^+ *transporting subunit beta 3; Atp4b, ATPase* H^+/K^+ *transporting beta subunit; Atp6v1b2, ATPase* H^+ *transporting V1 subunit B2; Atpaf1, ATP synthase assembly factor 1; Atpaf2, ATP synthase assembly factor 2; Cables1, Cdk5 and Abl enzyme substrate 1; Cacna1c, Calcium voltage-gated channel subunit alpha1 C; Cacna1d, Calcium voltage-gated channel subunit alpha1 D; Cacna2d3, Calcium voltage-gated channel auxiliary subunit alpha2delta 3; Cacnb2, Calcium voltage-gated channel auxiliary subunit beta 2; Camk1d, Calcium/calmodulin-dependent protein kinase 1d; Camk2a, Calcium/calmodulin-dependent protein kinase II alpha; Camk2d, Calcium/calmodulin-dependent protein kinase II delta; Cav1, Caveolin 1; Cav2, Caveolin 2; Cav3, Caveolin 3; Cdc42ep4, CDC42 effector protein 4; Cdh2, Cadherin 2; Cdh20, Cadherin 20; Cds1, CDP-diacylglycerol synthase 1; Cds2, CDP-diacylglycerol synthase 2; Cfl2, Cofilin 2; Creb3l2, CREB3-like transcription factor 2; Des, Desmin; Ednra, Endothelin receptor type A;* ERK, Extracellular signal-regulated kinase*; Fasn, Fatty acid synthase; Gna13, G protein subunit alpha 13; Gng12, G protein subunit gamma 12; Gng2, G protein subunit gamma 2; Gng4, G protein subunit gamma 4; Gpd1, Glycerol-3-phosphate dehydrogenase 1; Gpd2, Glycerol-3-phosphate dehydrogenase 2; Grb2; Growth factor receptor bound protein 2; Gys2, Glycogen synthase 2; Hspb2, Heat shock protein family B member 2; Il15, Interleukin 15; Itga8, Integrin subunit alpha 8; Itgb6, Integrin subunit beta 6; Itpr1, Inositol 1,4,5-trisphosphate receptor type 1; Itpr2, Inositol 1,4,5-trisphosphate receptor type 2; Itpr3, Inositol 1,4,5-trisphosphate receptor type 3; Kcna4, Potassium voltage-gated channel subfamily A member 4; Kcnab1, Potassium voltage-gated channel auxiliary subfamily B member 1; Kcnb1, Potassium voltage-gated channel subfamily B member 1; Kcng4, Potassium voltage-gated channel modifier subfamily G member 4; Kcnh2, Potassium voltage-gated channel subfamily H member 2; Kcnj3, Potassium inwardly-rectifying channel subfamily J member 3; Kcnk3, Potassium two-pore domain channel subfamily K member 3; Kcnq1, Potassium voltage-gated channel subfamily Q member 1; Lama2, Laminin subunit alpha 2;* MAPK, Mitogen-activated protein kinase*; Mapk12, Mitogen-activated protein kinase 12; Mapk4, Mitogen-activated protein kinase 4; Mlxipl, MLX interacting protein-like (ChREBP); Nd1, NADH dehydrogenase*

subunit 1 (mitochondrial); Nd2, NADH dehydrogenase subunit 2; Nd3, NADH dehydrogenase subunit 3; NAFLD, Non-alcoholic fatty liver disease*; Nd4, NADH dehydrogenase subunit 4; Nd4l, NADH dehydrogenase subunit 4L; Nd5, NADH dehydrogenase subunit 5; Nd6, NADH dehydrogenase subunit 6; Nfatc1, Nuclear factor of activated T cells 1; Nfatc2, Nuclear factor of activated T cells 2; Nfatc3, Nuclear factor of activated T cells 3; Nfatc4, Nuclear factor of activated T cells 4; Ntrk2, Neurotrophic receptor tyrosine kinase 2; Pak6, p21-activated kinase 6; Phkg1, Phosphorylase kinase catalytic subunit gamma 1; Pla2g5, Phospholipase A2 group V; Plcb1, Phospholipase C beta 1; Pln, Phospholamban; Pnpla3, Patatin-like phospholipase domain containing 3; Ppm1j, Protein phosphatase* Mg^{2+}/Mn^{2+} *dependent 1J; Ppp1ca, Protein phosphatase 1 catalytic subunit alpha; Prkag2, Protein kinase AMP-activated non-catalytic subunit gamma 2; Prkar1a, Protein kinase A regulatory subunit 1A; Prkar2b, Protein kinase A regulatory subunit 2B; Prkca, Protein kinase C alpha; Prkcb, Protein kinase C beta; Ptpmt1, Protein tyrosine phosphatase mitochondrial 1; Rhoa, Ras homolog family member A; Rhob, Ras homolog family member B; Rhoj, Ras homolog family member J; Rhou, Ras homolog family member U; Rock1, Rho-associated protein kinase 1; Rock2, Rho-associated protein kinase 2; Ryr2, Ryanodine receptor 2; Scn1b, Sodium voltage-gated channel beta subunit 1; Scn2b, Sodium voltage-gated channel beta subunit 2; Scn3b, Sodium voltage-gated channel beta subunit 3; Scn4b, Sodium voltage-gated channel beta subunit 4; Slc25a4, Solute carrier family 25 member 4; Slc25a5, Solute carrier family 25 member 5; Slc25a6, Solute carrier family 25 member 6; Slc8a1, Sodium/calcium exchanger 1; Srebf1, Sterol regulatory element–binding transcription factor 1; Stat3, Signal transducer and activator of transcription 3; Stat5b, Signal transducer and activator of transcription 5B; Tpcn2, Two-pore segment channel 2; Trdn, Triadin*

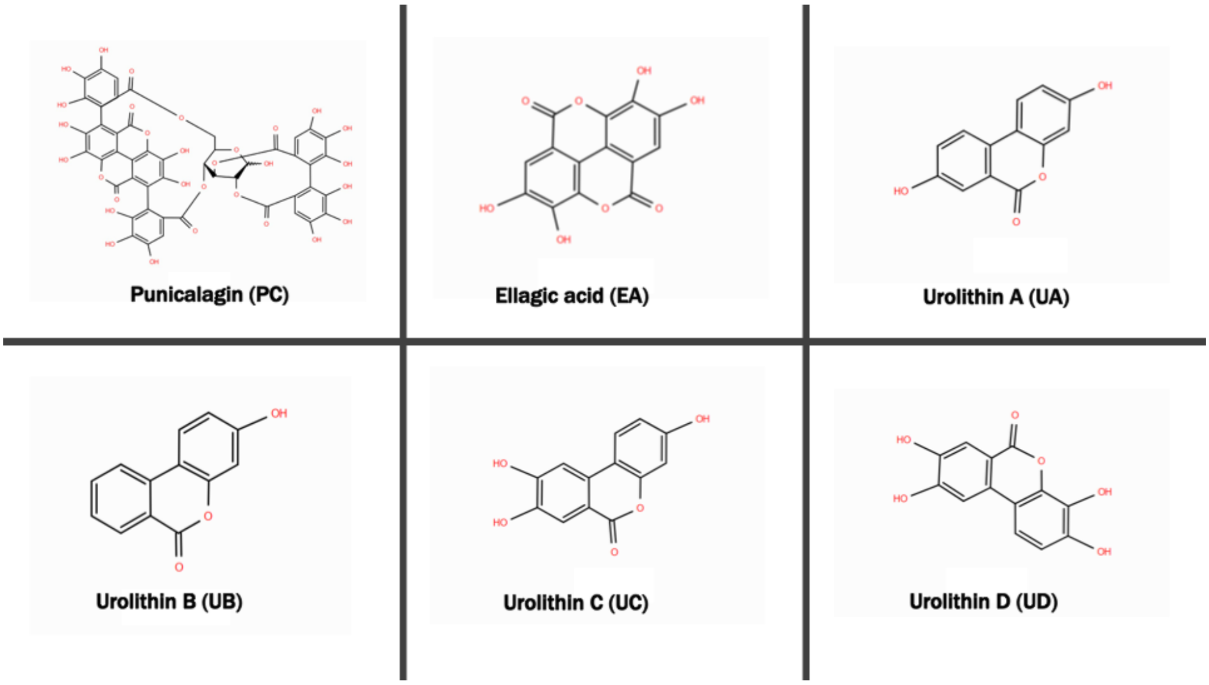

Supplementary Figure S1. Structures of punicalagin, ellagic acid and urolithins A-D

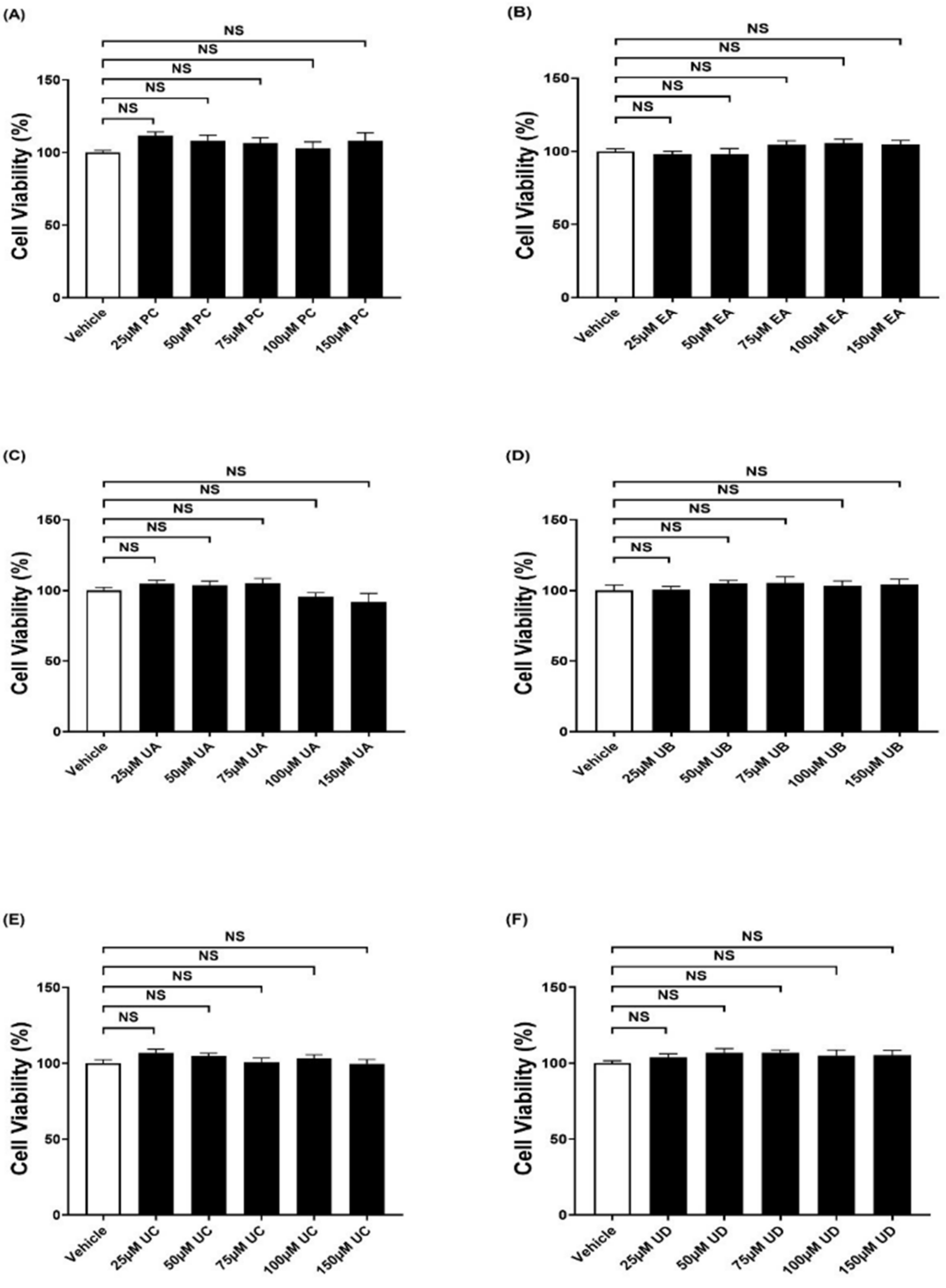

Supplementary Figure S2. PC and its metabolites have no effects on the viability of THP-1 macrophages after 24 hours

THP-1 macrophages were treated for 24 h with the DMSO vehicle (vehicle control) or different concentrations (25 μM, 50 μM, 75 μM, 100 μM and 150 μM) of PC (A) or its metabolites: EA (B), UA (C), UB (D), UC (E) and UD (F) as indicated. Cell viability was assessed by monitoring the lactate dehydrogenase levels in the supernatant. The results are presented as percentage (mean ± SEM) of the vehicle control (assigned 100%) from four independent experiments. Statistical analysis was carried out by one-way ANOVA with Dunnett post-hoc analysis (NS, not significant).

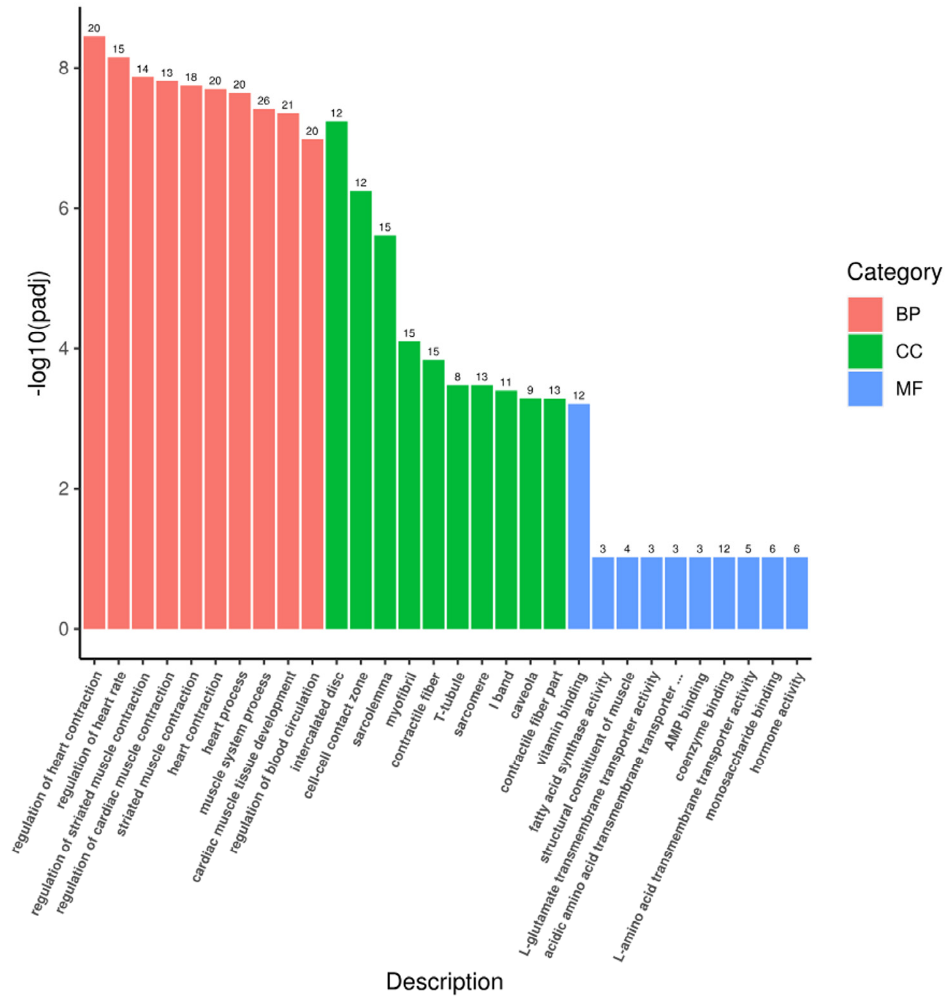

Supplementary Figure S3. The most enriched Gene Ontology (GO) terms bar chart

The GO term enrichment analysis is categorised into three main GO terms: Biological Process (BP); Cellular Component (CC) and Molecular Function (MF). The y-axis represents the negative logarithm of the p-value (adjusted for false discovery rate) on a base 10 scale (-log10 [p-adj]), indicating the significance of enrichment. Each bar represents a different GO term, as labelled on the x-axis, with the bar's height reflecting the enrichment significance of that term. The bars are colour-coded: red for BP, green for CC, and blue for MF. Numbers atop the bars represent the count of genes associated with each GO term.

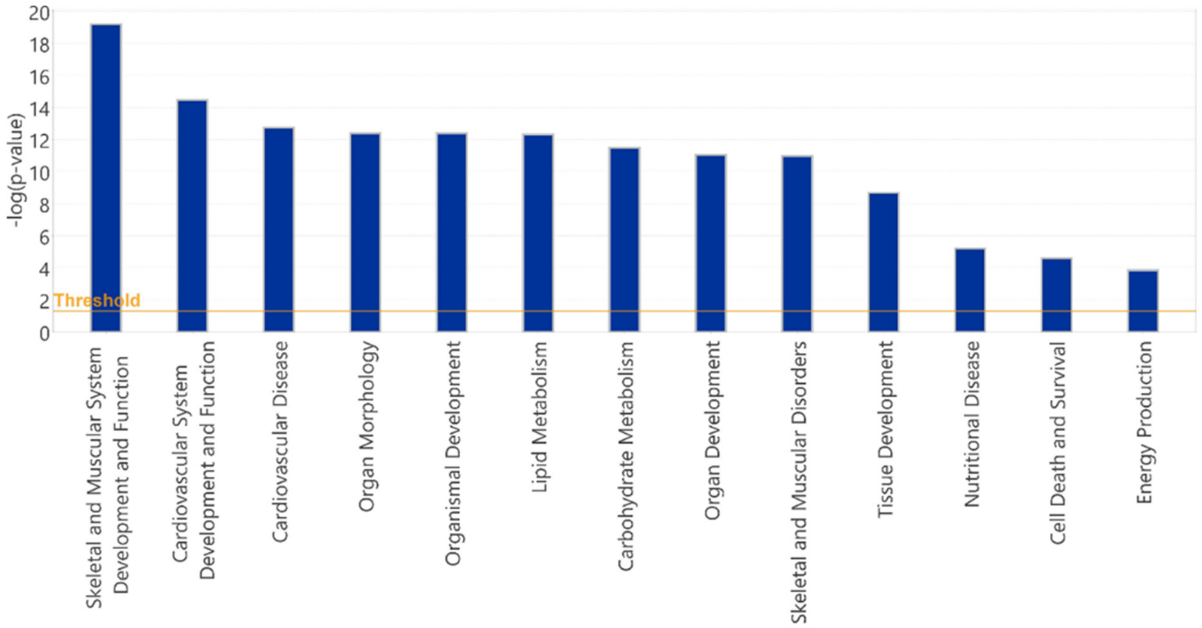

Supplementary Figure S4. Key biological functions altered by UA.
The figure illustrates key biological functions affected by UA treatment, with a focus on pathways related to atherosclerosis. The bars represent the -log(p-value), indicating the statistical significance of the impact on each biological function, the longer the bar, the more significant the impact. The threshold line represents the default significance level (p-value of 0.05), with functions surpassing this line considered as statistically significant.
